# Interspecific selection in a diverse mycorrhizal symbiosis

**DOI:** 10.1038/s41598-024-62815-4

**Published:** 2024-05-27

**Authors:** Megan A. Rúa, Jason D. Hoeksema

**Affiliations:** 1 Department of Biological Sciences, Wright State University, 3640 Colonel Glenn Hwy, Dayton, OH 45435 USA; 2 Department of Biology, University of Mississippi, P.O. Box 1848, University, MS 38677 USA

**Keywords:** Community ecology, Ectomycorrhizal fungi, Evolution, Mutualism, Soil micro-organisms, Symbiosis, Coevolution, Microbial ecology

## Abstract

Coevolution describes evolutionary change in which two or more interacting species reciprocally drive each other’s evolution, potentially resulting in trait diversification and ecological speciation. Much progress has been made in analysis of its dynamics and consequences, but relatively little is understood about how coevolution works in multispecies interactions, i.e., those with diverse suites of species on one or both sides of an interaction. Interactions among plant hosts and their mutualistic ectomycorrhizal fungi (ECM) may provide an ecologically unique arena to examine the nature of selection in multispecies interactions. Using native genotypes of Monterey pine (*Pinus radiata*), we performed a common garden experiment at a field site that contains native stands to investigate selection from ECM fungi on pine traits. We planted seedlings from all five native populations, as well as inter-population crosses to represent intermediate phenotypes/genotypes, and measured seedling traits and ECM fungal traits to evaluate the potential for evolution in the symbiosis. We then combined field estimates of selection gradients with estimates of heritability and genetic variance–covariance matrices for multiple traits of the mutualism to determine which fungal traits drive plant fitness variation. We found evidence that certain fungal operational taxonomic units, families and species-level morphological traits by which ECM fungi acquire and transport nutrients exert selection on plant traits related to growth and allocation patterns. This work represents the first field-based, community-level study measuring multispecific coevolutionary selection in nutritional symbioses.

## Introduction

Coevolution describes evolutionary changes in which two or more interacting species reciprocally alter each other’s evolution, but the importance of coevolutionary processes for shaping evolutionary diversification has been an area of debate within evolutionary biology, especially for interactions involving numerous species^1–4^. Studies have shown that interspecific selection—selection by one or more species on the traits of another species—can lead to ecological speciation by driving adaptive differentiation among populations, which can lead to sustained evolutionary change in species at multiple spatial and temporal scales^5^. Thus, studies of interspecific selection can provide insight into the fundamental processes generating and maintaining biodiversity, including genetic and phenotypic diversity within and between species. Such studies also represent building blocks in our understanding of coevolution. Indeed, for most species interactions and especially for diverse multi-species interactions, interspecific selection has rarely been measured in one direction, much less reciprocally^6^.

Analyzing interspecific selection and coevolution in diverse multi-species interactions (multispecific coevolution) presents a unique challenge because there exists a wide range of scenarios both for the number of traits involved and for the degree of independence of evolutionary dynamics in any particular species pair that makes it difficult to tease apart directionality and sources of selection. For example, in an interaction between a single plant host and a diverse guild of herbivores, the outcome of interspecific selection may vary along a continuum such that host plant traits all experience selection from the herbivore community in the same direction or those same host plant traits may experience selection from individual herbivores within the same community in different directions. Additionally, different herbivore species may also alter each other’s evolutionary dynamics with the host. This entire multispecific coevolution process has been called diffuse coevolution and has received very little empirical attention despite the ecological importance of multispecies communities^7,8^. The empirical attention it has received has come from antagonistic interactions, such as plant–herbivore systems (e.g.,^2,9–12^). These studies have sometimes found that genetic covariance among host traits constrain evolutionary responses of the host to diverse antagonists such that hosts are never able to fully obtain maximum fitness^11^.

Interspecific selection in mutualisms may operate differently than in antagonistic systems, and may also depend on whether the mutualism is symbiotic or free-living. Evidence to date suggests that symbiotic multispecific mutualisms may evolve to favor complementary sets of non-competing symbionts, while free-living (non-symbiotic) multispecific mutualisms may evolve to favor the accumulation of species that share a core set of mutualistic traits, rather than specializing on a partner species or mode of interacting with the host^13^. Individual plants are often concurrently associating with diverse rhizosphere microorganisms^14,15^ such as mycorrhizal fungi, which are common symbionts of over 80% of terrestrial plants^16^. Because mycorrhizal symbioses are diverse, multispecies interactions in which multiple fungi can associate with the same plant and vice versa^17,18^, it is not clear whether we expect them to evolve towards a core set of shared mutualistic traits (as predicted for purely free-living mutualisms) or towards a set of complementary non-competing symbionts that have unique modes of interaction with each other (as predicted for intimate symbiosis^13^). Experiments with pines and ectomycorrhizal (ECM) fungi suggest that pine populations have evolved preferences for particular fungal species^19,20^ and that some of these interactions may be controlled in plants by independent loci of large effect^21^. Estimates of natural selection by ECM fungi on plant traits could lend insight into how interspecific selection may operate in such multispecific mutualistic species interactions, yet we lack direct field estimates of natural selection in these diverse interactions.

Monterey Pine (*Pinus radiata* D.Don) is a locally dominant conifer, the native range of which consists of small, isolated populations spanning a broad latitudinal gradient^22^. Post-Pleistocene native populations of Monterey pine are restricted to a small set of geographically separated sites along the west coast of California (USA) and two islands off Baja California (Mexico)^23^. The geographic isolation of these populations^24^ provides an opportunity to study how isolated sets of diverse interactions evolve in different contexts. Moreover, the native populations of Monterey pine not only harbor different communities of ECM fungi^25^, and also exhibit significant genetic differentiation in compatibility with particular species of ECM fungi and in several growth/allocation traits, including growth rate, biomass allocation among shoots and roots, and root coarseness^19,20^. However, it is unknown whether and how natural selection, including interspecific selection from ECM fungi, may have driven the diversification of those traits. A field experiment where plants have access to a wider array of ECM fungi than in a greenhouse inoculation experiment could be used to test that hypothesis.

In order to directly estimate the effect of phenotypic variation on plant fitness in these interactions, we conducted a common garden experiment in which seedlings from *P. radiata* phenotypes representing a broad suite of possible plant traits were grown in a single location, minimizing the influence of environmental variation on plant fitness. We first used traditional quantitative genetic approaches to evaluate the extent to which plant and fungal traits are heritable. We then estimated selection gradients of plant and fungal traits on three proxies for plant fitness using traditional selection analysis^26^ for relative growth rate and total biomass and using logistic selection analysis^27^ for plant survival.

## Methods

### Field experiment and measured traits

The common garden experiment was conducted at the University of California at Santa Barbara’s Kenneth S. Norris Rancho Marino Reserve (35.535, − 121.08) in Cambria, California, USA, where Monterey pine was the dominant ECM fungal tree species, although occasional coast live oaks (*Quercus agrifolia*) were observed.

So that natural selection in the experiment was constrained as little as possible by available phenotypes, we sought to establish the common garden with a broad range of Monterey pine traits (including compatibility with particular ECM fungi). To achieve this goal, the common garden was planted with seeds from open-pollinated maternal families of all three California populations (Año Nuevo, Monterey and Cambria), both Mexican populations (Cedros Island and Guadalupe Island), and progeny seeds from controlled crosses involving each of the five native populations (Supplementary Table S1; Supplementary Materials C). Prior to planting, seeds were surface-sterilized in 10% bleach for 5 min, rinsed thoroughly, and stratified at 4 °C for four weeks, at which point seeds were germinated in potting medium (Metro Mix 360; Sun Gro Horticulture Inc., Agawam, Massachusetts, USA) and grown for 6–8 weeks in a greenhouse at the University of Mississippi (34.358158, − 89.550439). Seedlings were carefully removed from the germinating flats, checked to confirm lack of ECM colonization, and shipped overnight to the field site in California. Seedlings that survived the shipment were planted May 1–2, 2013.

Overall, we planted 1178 seedlings from 47 families into the understory of a mixed size class Monterey Pine-dominated forest at the field site. Due to variation in germination rates in the greenhouse, replication for each family was variable, but most families had 30 seedlings (Supplementary Table S1). At planting, seedlings ranged in diameter 0.23–2.83 mm (average 1.07 mm; Supplementary Table S1) and height 1–15.5 cm (average 10 cm Supplementary Table S1), yielding a range of phenotypes. Seedlings were planted 15.25 cm (6 inches) apart and were randomly assigned into 30 rows with 40 seedlings per row, except the last row, which had 18 seedlings.

Seedlings were allowed to grow for 16 weeks, during which time survival and relative growth rate (RGR) were assessed. To estimate RGR we measured the length of the needle-bearing stem on each plant, as previous work in our system has indicated it is tightly correlated with total biomass^17^. RGR was estimated as [ln(*h*_*2*_)-ln(*h*_*1*_)]/(no. of days), where *h*_*1*_ is the length of needle-bearing stem at planting and *h*_*2*_ is the length of needle-bearing stem at harvest. At harvest (September 2013), we also measured the plant traits total biomass, root:shoot biomass allocation, and specific root length (SRL, meters per gram of root) on all surviving seedlings. Plant root length was estimated using a grid-intersect method^30^. Shoot and root biomass were separated, dried at 60 °C for 72 h, and weighed.

ECM fungal abundance and composition were estimated on surviving seedlings by counting the number of pine root tips colonized by each morphotype and then classifying each morphotype into operational taxonomic units (OTUs) using DNA sequencing (see Supplementary Materials C). Since colonization of a host root by a particular mycorrhizal fungus is likely influenced by plant genes, fungal genes, and the abiotic environment, the abundances and characteristics of mycorrhizal fungi on the root system of a plant can not only be viewed as traits of the mycorrhizal fungal community associated with the plant, but also as a "mycorrhizal trait" of that plant, related to the symbiotic compatibility of the plant with that fungal species^19,31^. From this perspective of community genetics, the fungal community is part of the extended phenotype of the plant^28^.

### Determination of fungal exploration types

Consensus fungal sequences from each OTU were checked using BLAST^29^ searches on the International Nucleotide Sequence Database (INSD) and the User-Friendly Nordic ITS Ectomycorrhizal (UNITE) database^30^ to obtain best matches for taxonomic affiliation of OTUs. Any species known to be strictly non-mycorrhizal was eliminated from the data set. More details on taxonomic assignment of sequences can be found in the Supplementary Materials C. The raw fungal sequence data for this project have been submitted to the GenBank databases under the accession numbers MN364462–MN364644.

When fungal OTUs could be identified to species, fungal traits associated with foraging strategy, foraging distance, rhizomorph formation, and hydrophobicity^31^ were assigned using the Determination of EctoMYcorrhiza database (DEEMY, http://www.deemy.de). Since foraging-related functional traits are typically conserved at the genus level^32^, when no species-level matches were available in DEEMY, entries for congeners associated with *Pinus* were surveyed and consensus trait values were assigned if 90% of entries agreed. This allowed for OTUs that could only be identified to genus to also be assigned trait values^33,34^. OTUs were categorized into traits associated with exploration type: contact, short, medium fringe, medium smooth, and long distance. These traits incorporate differences in mycelial growth pattern, extent of biomass accumulation, hydrophobicity of the hyphae, and the presence or absence of rhizomorphs as well as hyphal production categories (‘Low Biomass’ or ‘High Biomass’) as a function of rhizomorph production and extent of biomass accumulation^35^. These fungal traits were then transformed into plant quantitative trait values as a function of tree genetic family by dividing the number of identified mycorrhizal root tips per exploration type on each plant genetic family by the sum of tips from all identified OTUs on that plant genetic family.

#### Estimation of fungal richness and diversity

Alpha diversity (the ECM fungal diversity on the root system of a single seedling) was estimated using the Observed richness (number of observed OTUs), the Chao1 index which estimates species richness based on abundance distributions, the Shannon diversity index which integrates richness and evenness, and the inverse Simpson’s diversity index which also integrates richness and evenness but gives more weight to the more abundant species. The Chao1 index was calculated in R with the *estimateR* function in the *vegan* package^36^, while the Shannon and Simpson indices were both calculated with custom functions. Since the Observed richness/Chao1 and Simpson Index/Shannon Index measures of alpha diversity were highly correlated (Pearson’s correlations p < 0.0001, Supplementary Table S2C), only the Observed and Shannon index results are presented in the main manuscript and the Chao1 and Simpson index are reported in the Supplementary Material.

#### Statistical analyses

First, we used quantitative genetics to calculate measures of heritability in the plants for both explicitly "plant traits" (root:shoot, diameter, RGR, biomass), explicitly "fungal traits" (fungal exploration types and hyphal biomass), as well as for potential extended phenotypes of the plant related to their ECM fungal symbionts (relative abundances of particular OTUs or families, or fungal diversity metrics). We refer to the latter as "mycorrhizal traits" for simplicity, since their trait values on a plant may be influenced by both plant genes (extended phenotype of the plant) and fungal genes. We then evaluated the potential for both constraint and facilitation in the evolution of mycorrhizal relationships using the genetic-covariance matrix for plant, fungal, and mycorrhizal traits. Finally, we used logistic selection analysis^27^ and traditional selection analysis^26^ to estimate selection gradients of plant, fungal, and mycorrhizal traits on quantitative proxies for plant fitness including biomass, RGR, and survival. We also used logistic regression with plant survival (proportion survived per genotype) as the response to test for evidence of local adaptation by the Cambria population, adaptation by mainland versus island populations, differences between the island populations, and advantage of hybrid versus single population genetic background. Further details on analysis methodology can be found in Supplementary Material C: Methods. All analyses were done with R statistical software, version 4.3.0^37^, and models are outlined in Supplementary Material A. Unless noted, all figures were created with ggplot2^38^.

## Results

### Overall plant survival

Out of the 1178 seedlings, 472 survived (Supplementary Table S1). The odds of mortality were 1.23 times greater with a pure genetic background compared to that of a hybrid (P = 0.078); however, the odds of mortality with a pure island background (P = 0.0157) or hybrid between an island and mainland pine (P = 0.0095) were lower (0.70 and 0.69 times) than the odds of surviving as a pure mainland pine. There was no significant difference in odds of survival between pines with a Cambria background compared to pines without a Cambria background (P = 0.1688) or between pines with a Cedros background compared to pines with a Guadalupe background (P = 0.2167).

### Plant traits

Our analysis revealed a substantial amount of genetic variation in plant traits. The estimated heritability (h^2^) for biomass, diameter, RGR and SRL was all relatively high (Supplementary Table S8) while the heritability for root:shoot was only low to moderate (h^2^ = 0.06 (95% CI 0.008, 0.15)). When biomass and RGR were used as plant fitness proxies, there was total selection for increased RGR or plant biomass (in the biomass and RGR models, respectively), diameter, and decreased SRL (Supplementary Material A, Supplementary Fig. S4). Root:shoot experienced significant total selection across all plant fitness models but the pattern differed such that selection was negative when RGR and biomass were used as plant fitness proxies but positive when the proportion survived was used as a fitness proxy (Supplementary Material A, Supplementary Fig. S4).

### Fungal and mycorrhizal traits

i. OTUs

The ECM fungal community was identified from a total of 11,211 colonized root tips, and consisted of 66 OTUs from 20 families (Supplementary Fig. S2A). Heritability for overall tip abundance was relatively low (h^2^ = 0.013 (95% CI 0.0, 0.04)), but heritability for many individual OTUs and families was much higher (Supplementary Table S8). The five most common fungal families were Thelephoraceae (66% of total tips), Russulaceae (14% of total tips), Sebacinaceae (5% of total tips), Gloniaceae (4% of total tips), and Atheliaceae (3% of total tips) and the top five most abundant OTUs were *Cenococcum*, *Russula californiensis*, *Sebacinaceae1*, *Thelephoraceae1*, and *Tomentella1*.

**Figure 1:**
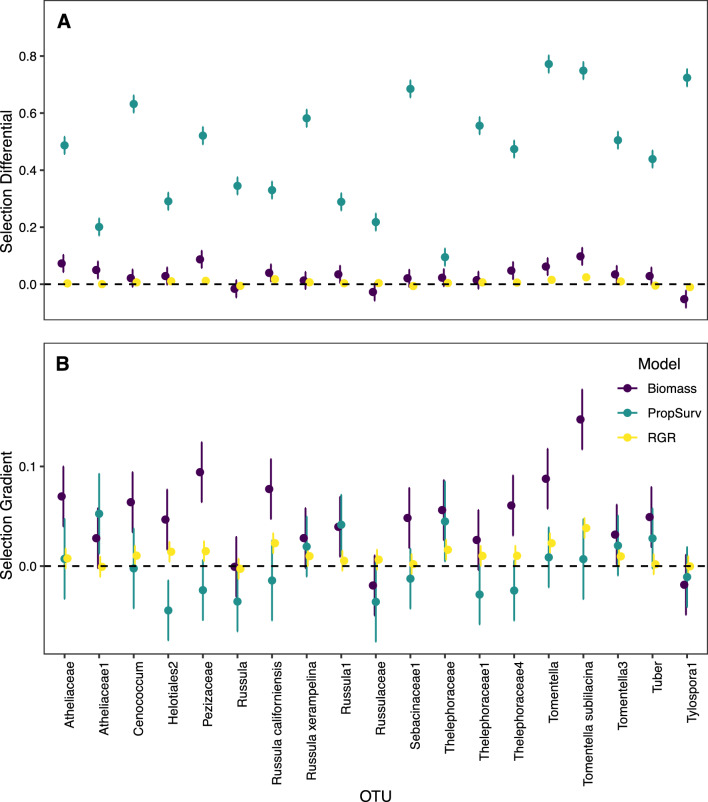
Results (estimate ± standard error) of selection analysis for selection differentials (**A**) and linear selection gradients (**B**) for operation taxonomic unit (OTU) models where plant biomass (purple), plant relative growth rate (RGR, yellow), and plant survival (teal) are used as plant fitness proxies (Supplementary Material A: Model 3). The x-axis for both plots represents values by OTU. The black dotted line indicates zero.

All OTUs had measurable heritability (Supplementary Table S8), but only the OTUs Helotiales2, Thelephoraceae and Thelephoraceae4 had genetic variances significantly greater than zero (Supplementary Table S8). There was significant total selection for several fungal OTUs (Supplementary Material A: Model 3). When biomass was used as a plant fitness proxy, there was positive selection for Atheliaceae1 and Pezizaceae1 (Supplementary Table S5). When biomass or RGR were used as fitness proxies, *Tomentella1* and *Tomentella sublilacina* were subject to positive selection, and Helotiales2 was subject to negative selection, (Supplementary Table S5, Fig. 1). *R. californiensis* experienced differential selection based on fitness proxy; when RGR was used as a fitness proxy, total selection was negative but when biomass was used as a fitness proxy, selection was positive (Supplementary Table S5, Fig. 1).

At the family level, there was measurable heritability for fungi from all five major families: Atheliaceae (h^2^ = 0.112 (95% CI 0.04, 0.19)), Gloniaceae (h^2^ = 0.056 (95% CI 0.02, 0.10)), Russulaceae (h^2^ = 0.066 (95% CI 0.02, 0.12)), Sebacinaceae (h^2^ = 0.11 (95% CI 0.05, 0.18)), Thelephoraceae (h^2^ = 0.046 (95% CI 0.01, 0.09)). However, fungi from the family Thelephoraceae were the only family whose genetic variance was significant. There was positive selection for the Pezizaceae when biomass was used as a fitness proxy and for Russulaceae when RGR was used as a fitness proxy (Supplementary Table S5). Thelephoraceae experienced positive selection when RGR or biomass were used as fitness proxies (Supplementary Table S5). No OTUs or families were subject to statistically significant selection when proportion survived was used as a plant fitness proxy (Supplementary Table S5, Fig. 1).

ii. Exploration type

Of 11,211 colonized root tips, 11,062 tips were classified according to their fungal exploration type. The most common exploration types were medium distance smooth (79% of total tips) and short distance (20% of total tips, Supplementary Fig. S2B). All of the exploration types exhibited a high degree of heritability (h^2^ > 0.50; Table 1), but none of the exploration types exhibited significant genetic variances (Table 1).

**Table 1:** Genetic variance–covariance matrix (G-matrix) and heritability (h^2^) for ectomycorrhizal fungal exploration types and plant traits. Genetic variances are on the main diagonal and covariances are off-diagonal elements. All genetic variances are statistically significant at P < 0.05. Genetic covariances significant at P < 0.05 indicated in bold.

	h^2^ (95% CI)	Biomass	Diam	RGR	RS	Contact	Long distance	Medium distance fringe	Medium distance smooth	Short distance
Biomass	0.22 (0.11,0.37)	− 0.123	0.129	0.542	− 0.132	− 0.016	0.023	0.028	− 0.027	0.005
Diam	0.22 (0.09,0.36)		− 0.059	0.009	0.059	− 0.059	0.027	0.083	− 0.063	− **0.213**
RGR	0.264 (0.14,0.40)			− 0.154	− 0.13	− 0.059	0.003	0.059	− 0.008	**0.059**
RS	0.06 (0.008,0.15)				− 0.071	0.132	0.06	− 0.158	− 0.021	0.132
Contact	0.345 (0.17,0.53)					− 0.115	0.417	0.045	− 0.543	0.236
Long Distance	0.49 (0.27,0.71)						0.012	− 0.214	− 0.453	0.158
Medium Distance Fringe	0.321 (0.15,0.51)							− 0.117	− 0.232	− 0.375
Medium Distance Smooth	0.693 (0.46,0.91)								0.044	− **0.052**
Short Distance	0.73 (0.50,0.92)									− 0.094

*CI* credible interval, *R:S* Root (mg):Shoot (mg), *RGR* relative growth rate.

**Figure 2:**
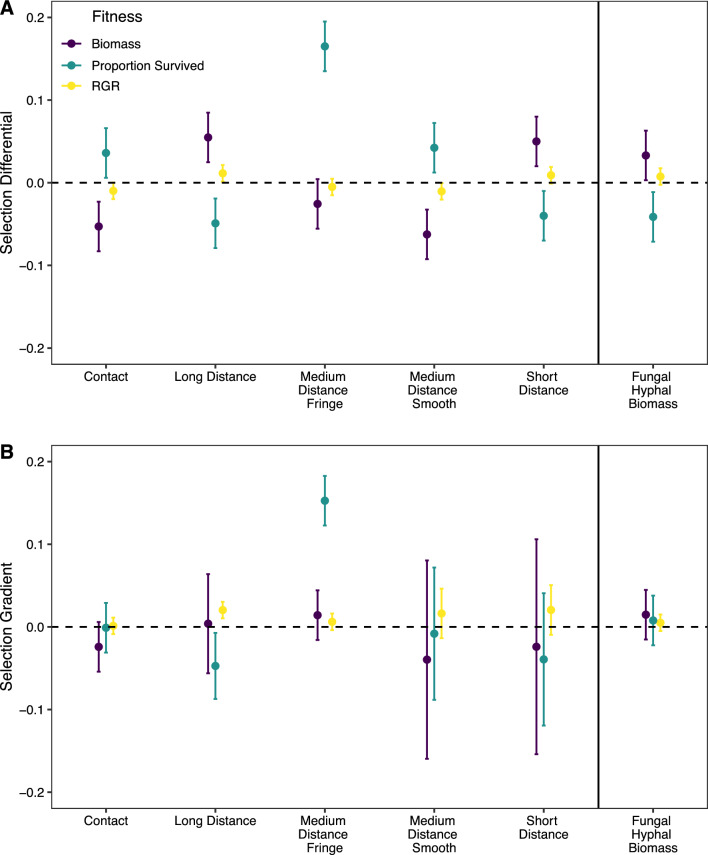
Results (estimate ± standard error) of selection analysis for selection differentials (**A**) and linear selection gradients (**B**) for exploration type and fungal hyphal biomass fitness models when biomass (purple), survival (teal) and relative growth rate (RGR, yellow) are used as proxies for plant fitness (Supplementary Material A: Model 4 and 5). Red dotted line indicates zero.

Only fungi from the contact, medium distance smooth, short distance and long distance exploration types experienced statistically significant selection. When RGR was used as a fitness proxy, there was selection for decreased abundance of fungi from the medium distance smooth exploration type (Supplementary Material A: Model 5; Supplementary Table S4, Fig. 2). When proportion survived was used as a fitness proxy, there was selection for decreased abundance of fungi from the contact and medium distance smooth exploration types and increased abundance of fungi from the long distance and short distance exploration types (Supplementary Material A: Model 5; Supplementary Table S4).

A vast majority of the fungal OTUs were characterized by low fungal biomass (99% of total tips) but the distribution of high versus low biomass OTUs varied by host genetic background (Supplementary Fig. S3) and was moderately heritable (h^2^ = 0.576 (95% CI 0.32,0.80)) with a significant genetic variance. However, fungal hyphal biomass did not experience significant selection for any of the fitness proxies (Supplementary Material A: Model 4; Supplementary Table S3, Fig. 2).

iii. Diversity Indices

Heritability of diversity indices was moderate (${h}_{Shannon}^{2}=0.109 (95\text{\% CI 0.05,0.18})$; ${h}_{Simpon{\prime}s}^{2}=0.093 (95\text{\% CI}:\text{ 0.03,0.16})$) to low (${h}_{Chao1}^{2}=0.07 (95\text{\% CI 0.02,0.13})$; ${h}_{Observerd}^{2}=0.07 (95\text{\% CI 0.02,0.13})$). We identified significant total and linear directional selection for increased alpha diversity for all indices (Supplementary Table S6) when RGR or biomass was used as a fitness proxy (Supplementary Material A: Model 6a-l). None of the diversity indices were subject to statistically significant selection when proportion survived was used as a fitness proxy (Supplementary Table S6).

### Interspecific, plant-fungal selection

We assessed the potential for interspecific selection of ECM fungi on Monterey pine by examining interaction terms that combined plant traits and fungal traits. These terms represent selection on the plant trait resulting from its covariance with the fungal trait (Hoeksema 2010). We inferred the potential for interspecific selection when these interaction terms were significant.

i. Covariance of plant traits with total ECM tip abundance

There was significant positive selection for root:shoot and plant biomass due to ECM tip abundance when RGR was used as a fitness proxy (Supplementary Material A: Model 1b; Supplementary Table S7). When the proportion survived was used as a fitness proxy, RGR tended to experience negative selection due to ECM tip abundance (Supplementary Material A: Model 1c; Supplementary Table S7). None of the plant traits experienced significant selection from tip abundance when biomass was used as a fitness proxy (Supplementary Table S7).

ii. Covariance of plant traits with ECM fungal exploration types

**Figure 3:**
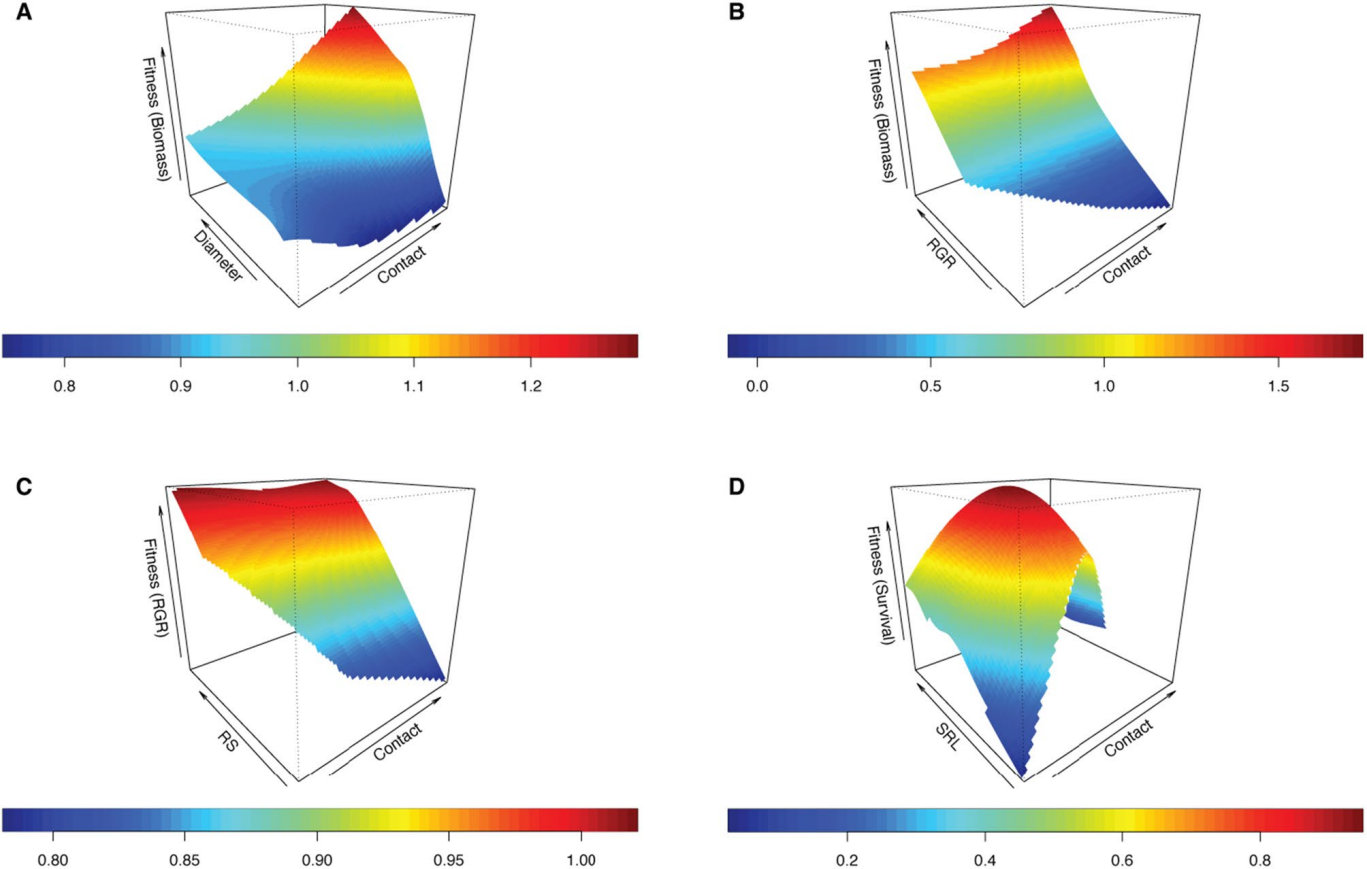
Fitness landscapes for co-evolutionary selection between fungi from the contact exploration type and (**A**) diameter and (**B**) relative growth rate (RGR) when biomass is used as a proxy for plant fitness, (**C**) root:shoot (RS) when RGR is used as a proxy for plant fitness and (**D**) specific root length (SRL) when survival is used as a plant fitness proxy. Heat colors indicate (**A**,**B**) relative biomass (effective df: A, 333.9; B, 332.8), (**C**) relative RGR (effective df: 334), and (**D**) survival probabilities (effective df: 7.4) estimated from thin-plate splines fit to the data by generalized cross-validation.

Fungi from the short distance exploration type exhibited significant genetic covariances with diameter and RGR (Table 1) which lead to positive significant selection for RGR (when biomass was used as a fitness proxy) and negative directional selection (when proportion survived was used as fitness proxy; Supplementary Table S4). RGR was also subject to negative directional selection due to the medium distance smooth (biomass only), contact (biomass only), and long distance exploration types (biomass and proportion survived; Supplementary Table S4). The fitness surface for RGR due to fungi from the contact exploration type is characterized by increases in peak relative biomass with increasing RGR and abundance of contact exploration type (Supplementary Material A: Model 5b; Fig. 3B).

**Figure 4:**
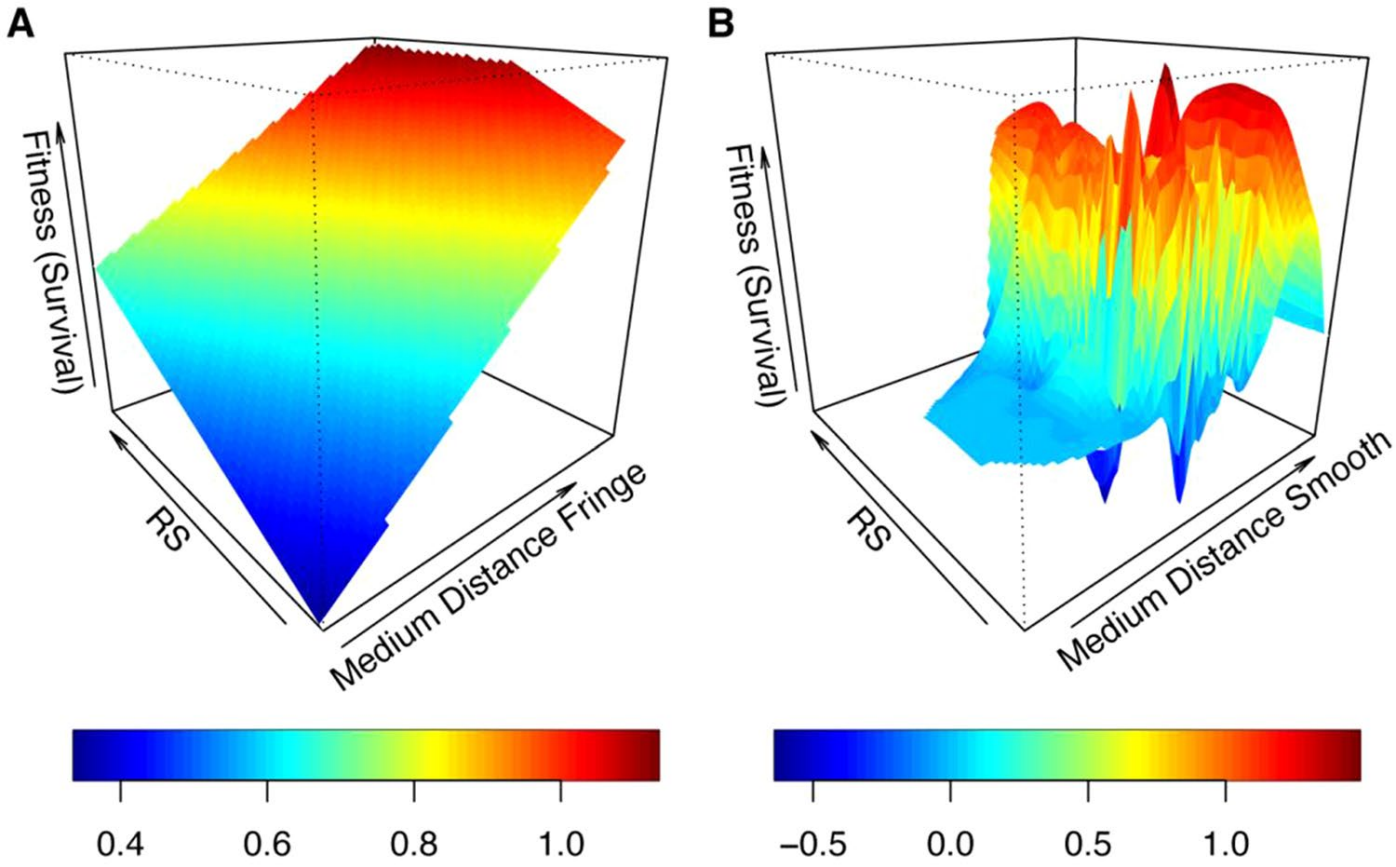
Fitness landscapes for co-evolutionary selection between fungi from (**A**) the medium distance fringe exploration type or (**B**) the medium distance smooth exploration type and root:shoot ratio (R:S) when survival is used as a plant fitness proxy. Heat colors indicate survival probabilities estimated from thin-plate splines fit to the data by generalized cross-validation (effective df: A, 336; B, 103.4).

Despite not identifying significant genetic covariances for other trait interactions, several plant traits experienced statistically significant selection due to their covariance with ECM fungal exploration types (Supplementary Material A: Model 5; Supplementary Table S4). In biomass models, diameter was subject to negative total selection with the contact exploration type (Supplementary Material A: Model 5a; Supplementary Table S4). The fitness surface for this relationship is characterized by a single fitness peak with decreasing trait values in all directions (Fig. 3A). Root:shoot was subject to positive total selection due to fungi from the medium distance fringe and medium distance smooth exploration types when proportion survived was used as a fitness proxy (Fig. 4); when RGR was used as fitness proxy, root:shoot experience negative total selection due to the contact exploration type and negative directional selection due to the contact, medium distance smooth, and short distance exploration types (Supplementary Table S4). SRL experienced statistically significant positive total selection due to fungi from the contact and medium distance fringe exploration types when survival was used as a fitness proxy (Supplementary Table S4; Fig. 3C). It was also subject to positive directional selection due fungi from the long distance exploration type for models where biomass was used as a fitness proxy (Supplementary Material A: Model 5a).

**Figure 5:**
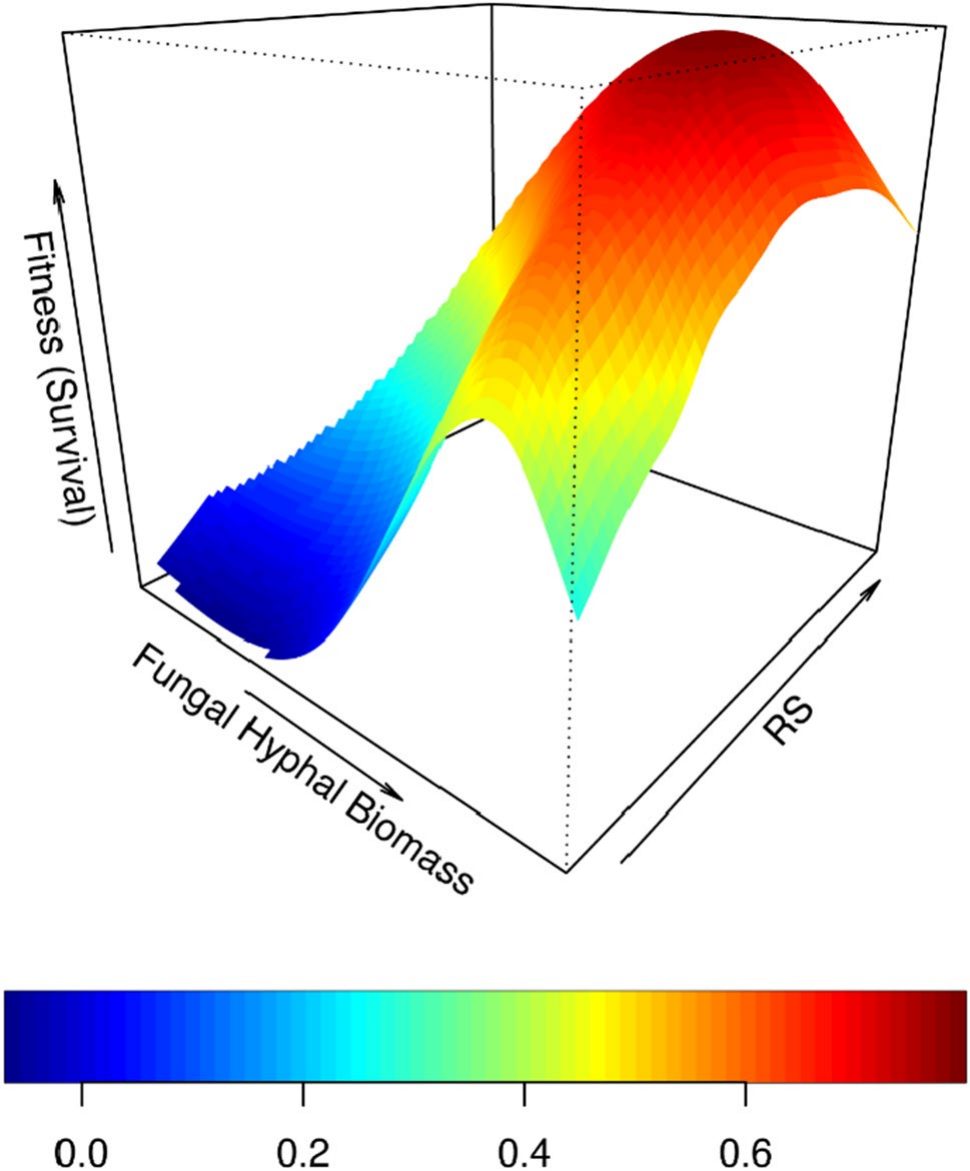
Fitness landscapes for co-evolutionary selection between fungal hyphal biomass and root:shoot ratio (R:S) when survival is used as a plant fitness proxy. Heat colors indicate survival probabilities estimated from thin-plate splines fit to the data by generalized cross-validation (effective df: 8.8).

Across fitness models, significant total selection was only detected for plant traits due to fungal hyphal biomass when survival was used as a fitness proxy (Supplementary Material A4c: Supplementary Table S3). Root:shoot was subject to positive total selection such that the fitness surface is characterized by a single fitness peak with decreasing trait values in all directions (Supplementary Table S3; Fig. 5). Diameter was subject to negative directional selection when RGR was used as a fitness proxy (Supplementary Table S3).

iii. Covariance of plant traits with abundance of dominant fungal *OTUs* & families

In G-matrices for the most abundant fungal OTUs and with all OTUs, several covariances between specific OTUs and plant traits were significant (Supplementary Table S8), leading to several instances of statistically significant total selection between plant traits and specific fungal OTUs or families (Supplementary Material A: Model 2, Table 2). When biomass and RGR were used as a fitness proxy, root:shoot was subject to positive total selection due to fungi from the OTU *Cenococcum,* and family Gloniaceae (Supplementary Material Model 2a–c,k–m: Table 2). Root:shoot was also subject to positive total selection due to fungi from the OTU *R. californiensis* when RGR was used as a fitness proxy (Supplementary Material A: Model 2b,f). SRL was subject to negative total selection due to fungi from the OTU *Tomentella1* when RGR was used as a fitness proxy (Supplementary Material A: Model 2b,f) and positive total selection due to the fungal family Sebacinaceae when biomass was used as a fitness proxy (Supplementary Material A: Model 2e; Table 2B). No plant traits experienced statistically significant total selection due to specific OTUs or families when survival was used as a fitness proxy.

**Table 2:** Results (model estimates ± SE) of selection analysis for selection differentials (*S*) and gradients (*β*) on plant and fungal traits and the plant trait resulting from its covariance with the fungal trait for (a) the six most abundant fungal OTUs and (b) five most abundant fungal families. Proportion Survived, Biomass, or Relative Growth Rate (RGR) were used as a proxy for Plant Fitness (Supplementary Material A: Model 4). *RGR* relative growth rate, *SRL* specific root length.

Trait	Source of selection	Fitness					
		Proportion survived		Biomass		RGR	
		Estimate	P-value	Estimate	P-value	Estimate	P-value
**(A)**							
Root:Shoot	*S*	0.0671 ± 0.04	0.0718	− 0.1002 ± 0.04	0.0051**	− 0.0309 ± 0.01	0.0001***
	*β*	0.1621 ± 0.05	0.0013**	− 0.0663 ± 0.04	0.0848	− 0.0229 ± 0.01	0.0117*
Diameter	*S*	0.0116 ± 0.04	0.7480	0.0861 ± 0.04	0.0163*	0.0171 ± 0.01	0.0349*
	*β*	0.0456 ± 0.04	0.1461	0.0245 ± 0.03	0.4285	0.0059 ± 0.01	0.4406
SRL	*S*	− 0.0020 ± 0.04	0.9570	− 0.1541 ± 0.03	< 0.0001***	− 0.0310 ± 0.01	0.0001***
	*β*	0.0632 ± 0.05	0.1356	− 0.0862 ± 0.03	0.0135*	− 0.0048 ± 0.01	0.5751
RGR	*S*	− 0.0160 ± 0.04	0.6580	0.3152 ± 0.03	< 0.0001***	0.0710 ± 0.01	< 0.0001***
	*β*	0.0036 ± 0.05	0.9108	0.2239 ± 0.04	< 0.0001***	0.0553 ± 0.01	< 0.0001***
Cenococcum	*S*	− 0.0236 ± 0.04	0.5180	0.0445 ± 0.04	0.2170	0.0054 ± 0.01	0.5050
	*β*	0.0299 ± 0.05	0.8601	0.0831 ± 0.03	0.0148*	0.0017 ± 0.01	0.8346
x Root:Shoot	*S*	0.0482 ± 0.06	0.4340	0.1556 ± 0.06	0.0102*	0.0495 ± 0.01	0.0003***
	*β*	0.2109 ± 0.08	0.0100*	− 0.0347 ± 0.06	0.5354	0.0199 ± 0.01	0.1478
x Diameter	*S*	0.0242 ± 0.05	0.6510	0.0212 ± 0.05	0.6910	− 0.0031 ± 0.01	0.7960
	*β*	0.0644 ± 0.07	0.2089	0.0636 ± 0.05	0.1973	− 0.0125 ± 0.01	0.3156
x SRL	*S*	0.0397 ± 0.04	0.3760	− 0.0095 ± 0.04	0.8260	0.0002 ± 0.01	0.9840
	*β*	0.0884 ± 0.05	0.0182*	− 0.0159 ± 0.03	0.6433	0.0054 ± 0.01	0.5459
x RGR	*S*	− 0.0553 ± 0.04	0.1970	0.0381 ± 0.04	0.3560	0.0104 ± 0.01	0.3100
	*β*	− 0.1179 ± 0.05	0.1099	0.0064 ± 0.04	0.8631	0.0086 ± 0.01	0.4454
*Russula californiensis*	*S*	− 0.0407 ± 0.04	0.2710	0.0647 ± 0.04	0.0719	0.0180 ± 0.01	0.0264*
	*β*	0.0311 ± 0.06	0.7765	0.0742 ± 0.04	0.0797	0.0094 ± 0.01	0.3210
x Root:Shoot	*S*	− 0.0974 ± 0.07	0.1930	0.1431 ± 0.07	0.0522	0.0326 ± 0.02	0.0469*
	*β*	− 0.0111 ± 0.09	0.9836	− 0.0022 ± 0.06	0.9730	− 0.0140 ± 0.02	0.3747
x Diameter	*S*	− 0.0330 ± 0.05	0.5190	− 0.0012 ± 0.05	0.9800	0.0025 ± 0.01	0.8240
	*β*	0.0221 ± 0.07	0.8434	− 0.0413 ± 0.05	0.4001	0.0038 ± 0.01	0.7143
x SRL	*S*	0.0047 ± 0.06	0.9320	− 0.0385 ± 0.06	0.4850	0.0036 ± 0.01	0.7710
	*β*	− 0.0721 ± 0.07	0.1652	− 0.0829 ± 0.05	0.1062	0.0187 ± 0.02	0.2183
x RGR	*S*	− 0.0585 ± 0.04	0.1280	0.0129 ± 0.04	0.7160	0.0029 ± 0.01	0.7160
	*β*	− 0.1190 ± 0.07	0.1066	− 0.0167 ± 0.05	0.7353	0.0015 ± 0.01	0.8978
Thelephoraceae	*S*	0.0257 ± 0.03	0.1200	0.0444 ± 0.04	0.2190	0.0031 ± 0.01	0.7070
	*β*	0.0697 ± 0.05	0.0765	0.0733 ± 0.03	0.0330*	0.0077 ± 0.01	0.3705
x Root:Shoot	*S*	0.1018 ± 0.03	0.4310	− 0.0003 ± 0.03	0.9910	0.0056 ± 0.01	0.4240
	*β*	0.0128 ± 0.04	0.6623	− 0.0147 ± 0.03	0.5683	0.0039 ± 0.01	0.5404
x Diameter	*S*	0.0467 ± 0.05	0.1830	0.0496 ± 0.03	0.1100	0.0017 ± 0.01	0.8050
	*β*	0.0679 ± 0.04	0.0932	0.0243 ± 0.03	0.3542	− 0.0037 ± 0.01	0.6535
x SRL	*S*	0.0145 ± 0.05	0.7760	0.0108 ± 0.05	0.8320	0.0169 ± 0.01	0.1390
	*β*	0.1023 ± 0.06	0.0998	− 0.0267 ± 0.05	0.5662	0.0129 ± 0.01	0.2654
x RGR	*S*	− 0.0109 ± 0.06	0.8570	− 0.0997 ± 0.06	0.0969	− 0.0074 ± 0.01	0.3420
	*β*	− 0.0942 ± 0.07	0.1262	0.0287 ± 0.05	0.5716	− 0.0037 ± 0.01	0.7273
*Tomentella1*	*S*	0.0560 ± 0.04	0.8430	0.0895 ± 0.04	0.0125*	0.0157 ± 0.01	0.0532
	*β*	− 0.1213 ± 0.12	0.2371	0.0501 ± 0.08	0.5527	0.0192 ± 0.01	0.1853
x Root:Shoot	*S*	0.3024 ± 0.04	0.1980	0.0159 ± 0.04	0.6750	0.0073 ± 0.01	0.3910
	*β*	0.1245 ± 0.07	0.1813	− 0.0366 ± 0.05	0.4955	− 0.0062 ± 0.01	0.5407
x Diameter	*S*	− 0.0099 ± 0.01	0.5140	0.0237 ± 0.01	0.0635	0.0030 ± 0.01	0.3030
	*β*	0.0154 ± 0.03	0.7134	− 0.0235 ± 0.02	0.2944	− 0.0031 ± 0.01	0.5564
x SRL	*S*	− 0.0019 ± 0.04	0.9570	− 0.0748 ± 0.04	0.0350*	− 0.0104 ± 0.01	0.1940
	*β*	0.1574 ± 0.12	0.4272	− 0.0774 ± 0.09	0.3754	− 0.0131 ± 0.02	0.4916
x RGR	*S*	0.0063 ± 0.04	0.8700	0.0486 ± 0.04	0.2050	− 0.0046 ± 0.01	0.4110
	*β*	0.2229 ± 0.15	0.1500	0.0325 ± 0.11	0.7656	0.0190 ± 0.01	0.1399
*Tomentella sublilacina*	*S*	0.0019 ± 0.04	0.9580	0.1625 ± 0.03	< 0.0001***	0.0241 ± 0.01	0.0028**
	*β*	0.0104 ± 0.05	0.6742	0.1660 ± 0.03	< 0.0001***	0.0199 ± 0.01	0.0300*
x Root:Shoot	*S*	− 0.0252 ± 0.04	0.5640	− 0.0366 ± 0.04	0.4400	− 0.0044 ± 0.01	0.6530
	*β*	0.0226 ± 0.05	0.6190	− 0.0315 ± 0.04	0.3992	− 0.0044 ± 0.01	0.6206
x Diameter	*S*	0.0078 ± 0.04	0.8540	− 0.0151 ± 0.04	0.7210	− 0.0048 ± 0.01	0.6150
	*β*	0.0250 ± 0.05	0.5780	0.0111 ± 0.03	0.7447	− 0.0061 ± 0.01	0.4591
x SRL	*S*	0.0245 ± 0.04	0.5680	0.0378 ± 0.04	0.3760	0.0086 ± 0.01	0.3690
	*β*	0.0442 ± 0.05	0.2943	− 0.0008 ± 0.04	0.9829	0.0079 ± 0.01	0.3722
x RGR	*S*	0.0197 ± 0.04	0.6220	− 0.0089 ± 0.04	0.8230	− 0.0014 ± 0.01	0.8530
	*β*	− 0.0055 ± 0.05	0.8124	− 0.0183 ± 0.04	0.6126	− 0.0160 ± 0.01	0.0473*
**(B)**							
Root:Shoot	*S*	0.0660 ± 0.03	0.0547	− 0.0283 ± 0.02	0.0948	− 0.0312 ± 0.01	0.0001***
	*β*	0.1236 ± 0.04	0.0018**	− 0.0269 ± 0.02	0.2617	− 0.0174 ± 0.01	0.0241*
Diameter	*S*	0.0161 ± 0.03	0.6310	0.0494 ± 0.02	0.0033**	0.0184 ± 0.01	0.0142*
	*β*	0.0203 ± 0.04	0.3684	0.0779 ± 0.02	0.0007***	0.0062 ± 0.01	0.3423
SRL	*S*	− 0.0327 ± 0.03	0.3290	− 0.0770 ± 0.02	< 0.0001***	− 0.0318 ± 0.01	< 0.0001***
	*β*	0.0069 ± 0.04	0.9907	− 0.0247 ± 0.03	0.3243	− 0.0014 ± 0.01	0.1278
RGR	*S*	− 0.0098 ± 0.03	0.7690	0.1572 ± 0.01	< 0.0001***	0.0712 ± 0.01	< 0.0001***
	*β*	− 0.0060 ± 0.05	0.4190	0.1090 ± 0.03	< 0.0001***	0.0513 ± 0.01	< 0.0001***
Gloniaceae	*S*	− 0.0126 ± 0.03	0.7050	0.0117 ± 0.02	0.4910	0.0057 ± 0.01	0.4520
	*β*	0.0318 ± 0.04	0.7107	0.1249 ± 0.05	0.0060**	0.0026 ± 0.01	0.7149
x Root:Shoot	*S*	0.0484 ± 0.06	0.3980	0.0677 ± 0.03	0.0178*	0.0468 ± 0.01	0.0002***
	*β*	0.1967 ± 0.07	0.0111*	0.0248 ± 0.05	0.5852	0.0165 ± 0.01	0.1610
x Diameter	*S*	0.0159 ± 0.05	0.7430	0.0084 ± 0.02	0.7330	− 0.0039 ± 0.01	0.7230
	*β*	0.0495 ± 0.06	0.2885	0.0831 ± 0.04	0.0591	− 0.0102 ± 0.01	0.3605
x SRL	*S*	0.0562 ± 0.04	0.1990	0.0063 ± 0.02	0.7710	0.0015 ± 0.01	0.8690
	*β*	0.0888 ± 0.04	0.0144*	0.0324 ± 0.03	0.3483	0.0045 ± 0.01	0.5768
x RGR	*S*	− 0.0514 ± 0.04	0.1900	0.0048 ± 0.02	0.8060	0.0085 ± 0.01	0.3750
	*β*	− 0.1065 ± 0.05	0.0940	0.0414 ± 0.03	0.1341	0.0007 ± 0.01	0.4899
Russulaceae	*S*	− 0.0208 ± 0.03	0.5350	0.0213 ± 0.02	0.2080	0.0173 ± 0.01	0.0217*
	*β*	0.0155 ± 0.05	0.8774	0.0354 ± 0.04	0.3534	0.0181 ± 0.01	0.0364*
x Root:Shoot	*S*	− 0.0500 ± 0.04	0.2710	− 0.0039 ± 0.02	0.8610	0.0117 ± 0.01	0.2460
	*β*	− 0.0388 ± 0.05	0.4342	− 0.0611 ± 0.03	0.0766	− 0.0012 ± 0.01	0.8877
x Diameter	*S*	− 0.0003 ± 0.05	0.9950	− 0.0380 ± 0.02	0.1110	− 0.0112 ± 0.01	0.2900
	*β*	0.0381 ± 0.05	0.5621	0.0253 ± 0.03	0.4044	− 0.0002 ± 0.01	0.8219
x SRL	*S*	− 0.0486 ± 0.03	0.1550	− 0.0265 ± 0.02	0.1100	− 0.0044 ± 0.01	0.5450
	*β*	− 0.0447 ± 0.04	0.2697	0.0092 ± 0.03	0.7187	0.0093 ± 0.01	0.2577
x RGR	*S*	− 0.0291 ± 0.04	0.4270	− 0.0151 ± 0.02	0.4200	0.0019 ± 0.01	0.7880
	*β*	− 0.0922 ± 0.06	0.1000	− 0.0382 ± 0.04	0.2793	− 0.0061 ± 0.01	0.4912
Sebacinaceae	*S*	− 0.0139 ± 0.03	0.6790	0.0079 ± 0.02	0.6390	− 0.0100 ± 0.01	0.1860
	*β*	− 0.0379 ± 0.04	0.3726	− 0.1068 ± 0.10	0.2999	− 0.0069 ± 0.01	0.3499
x Root:Shoot	*S*	0.0147 ± 0.06	0.8000	− 0.0317 ± 0.03	0.2680	− 0.0030 ± 0.01	0.8200
	*β*	− 0.0539 ± 0.09	0.9750	− 0.0343 ± 0.05	0.5265	0.0160 ± 0.02	0.3786
x Diameter	*S*	− 0.0928 ± 0.05	0.0956	0.0031 ± 0.02	0.8970	0.0125 ± 0.01	0.2420
	*β*	− 0.0901 ± 0.09	0.6821	0.0571 ± 0.06	0.3490	0.0067 ± 0.01	0.5658
x SRL	*S*	− 0.0121 ± 0.04	0.7370	0.0087 ± 0.02	0.6520*	− 0.0054 ± 0.01	0.5060
	*β*	0.0103 ± 0.05	0.5285	− 0.0414 ± 0.06	0.5151	− 0.0058 ± 0.02	0.4712
x RGR	*S*	− 0.0572 ± 0.04	0.1850	− 0.0168 ± 0.02	0.4300	0.0007 ± 0.01	0.8990
	*β*	− 0.0385 ± 0.09	0.4024	− 0.0754 ± 0.10	0.4418	0.0033 ± 0.01	0.6870
Thelephoraceae	*S*	0.0010 ± 0.03	0.9750	0.0838 ± 0.02	< 0.0001***	0.0366 ± 0.01	< 0.0001***
	*β*	− 0.0004 ± 0.06	0.9328	0.0980 ± 0.03	0.0030**	0.0320 ± 0.01	0.0022**
x Root:Shoot	*S*	0.0417 ± 0.04	0.3190	− 0.0002 ± 0.02	0.9940	0.0145 ± 0.01	0.1210
	*β*	0.0511 ± 0.05	0.3335	0.0020 ± 0.02	0.9341	0.0051 ± 0.01	0.4982
x Diameter	*S*	− 0.0007 ± 0.02	0.9740	0.0095 ± 0.01	0.3860	0.0005 ± 0.01	0.9140
	*β*	− 0.0005 ± 0.02	0.7000	0.0175 ± 0.02	0.4353	− 0.0034 ± 0.01	0.3963
x SRL	*S*	0.0568 ± 0.03	0.1070	− 0.0037 ± 0.02	0.8370	− 0.0012 ± 0.01	0.8810
	*β*	0.0653 ± 0.06	0.1806	0.0070 ± 0.03	0.8270	0.0099 ± 0.01	0.2905
x RGR	*S*	− 0.0091 ± 0.03	0.7690	0.0093 ± 0.02	0.5630	0.0066 ± 0.01	0.2780
	*β*	0.0232 ± 0.05	0.9705	0.0109 ± 0.03	0.7123	− 0.0150 ± 0.01	0.0294*

***P < 0.001; **P < 0.01; *P < 0.05.

There were also a few instances of significant directional selection despite no evidence for statistically significant total selection. When biomass was used as fitness proxy, RGR experienced negative directional selection due to fungi from the OTU *T. sublilacina* and the family Thelephoraceae (Table 2) and, when survival was used as a plant fitness proxy, SRL and root: shoot experienced negative directional selection due to fungi from OTU *Cenococcum* and the family Gloniaceae (Table 2).

In G-matrices with the top five most abundant families, fungi from the family Russulaceae exhibited significant genetic covariances with plant biomass and RGR but there was no support for statistically significant selection for these fungi on any plant traits regardless of plant fitness proxy (Table 2).

iv. Covariance of plant traits with ECM fungal alpha diversity

Root:shoot, RGR, biomass and diameter all experienced statistically significant selection with measurements of ECM fungal alpha diversity (Supplementary Material A: Model 6, Supplementary Table S6). Due to covariance with the observed richness of ECM fungi, there was positive total selection for root:shoot when RGR was used as a fitness proxy (Supplementary Material: Model 6b: Supplementary Table S6), positive selection for diameter (Supplementary Material A: Model 6a: Supplementary Table S6) and positive directional selection for RGR when biomass was used as a fitness proxy when proportion survived was used as the fitness proxy (Supplementary Material: Model 6c: Supplementary Table S6).

**Figure 6:**
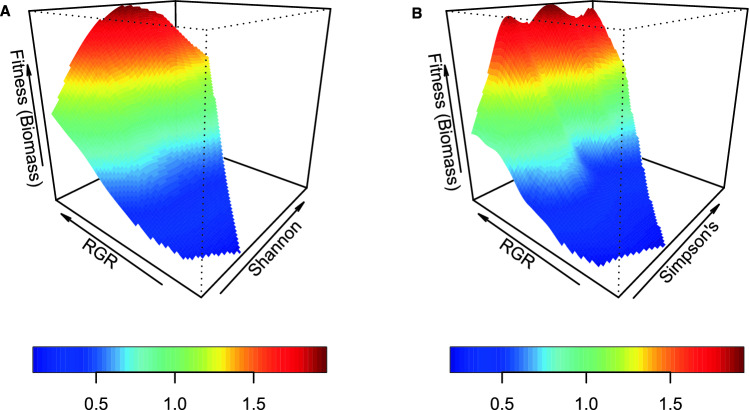
Fitness landscapes for co-evolutionary selection between plant relative growth rate (RGR) and ECM fungal (**A**) Shannon diversity and (**B**) Simpson’s diversity when biomass is used as a proxy for plant fitness. Heat colors indicate relative biomass estimated from thin-plate splines fit to the data by generalized cross-validation (effective df: A, 325.5; B, 307.2).

When biomass was used as a fitness proxy, there was significant total positive selection for RGR with the Shannon and Simpson’s diversity indices (Supplementary Material A: Model 6: Supplementary Table S6) such that peak relative biomass was reached with increasing RGR and Shannon diversity (Fig. 6). No plant traits measurements experienced statistically significant total selection with any of the diversity indices when survival was used as a fitness proxy.

## Discussion

In this study, we provide evidence for one side of coevolutionary selection in the diverse mutualism between ECM fungi and Monterey pine from a field study, i.e., selection of ECM fungi on Monterey pine. Despite the potential importance of coevolution for driving trait diversification^4,13^, there are relatively few examples that quantify reciprocal natural selection in diverse species interactions, i.e., multispecific coevolution. Instead, most studies quantify selection in pairwise host-parasite/predator/competitor interactions (i.e.,^11,39,40^). Indeed, we are not aware of any examples of studies estimating reciprocal selection forces in a diverse mutualism^6^. This paucity of evidence for coevolution in multispecies interactions may stem from the assumption that the complex nature of biotic selection in diverse interactions may prevent or override the effects of coevolution, making them difficult to measure^41^. It also may reflect limitations in the way coevolution has been traditionally defined, focusing on pairwise interactions; rather, it may be important to recognize that in diverse mutualisms, guilds (groups of species with similar traits) may have converged on core coevolving traits, and thus whole guilds may exert selection on another species in aggregate^6^. In addition, even if it may be difficult to measure responses to selection in individual members of diverse guilds, we suggest that analysis of guild-level traits can lend insight into how the traits of guilds of species may exert interspecific selection. As such, we considered how guild-level traits of the ECM fungal community, including their abundance, diversity, composition, and exploration morphology, may exert (as fungal traits) or respond to (as plant traits) selection on the plant. By combining field estimates of selection gradients with the genetic variance–covariance matrix for multiple traits of the mutualism, we found evidence that the presence of certain fungal OTUs, families, and exploration types can alter the evolutionary response of the plant to other mycorrhizal fungi.

### Selection on abundance of fungal *OTUs*

Fungal species within diverse assemblages of mycorrhizal fungi can be important sources of selection as individual fungal species may exert selective pressure on particular plant traits and plants may select for particular species of fungal taxa as well, influencing the resulting composition of the ECM fungal community. For example, plants may exhibit a degree of specificity in recognizing fungal partners by sanctioning or rejecting fungi if they are less beneficial^42,43^. While previous research with Monterey pine under controlled conditions has suggested that this species has evolved independently in response to different single species of ECM fungi^19,20^, here we show that in a field setting, multiple fungal OTUs and a single fungal family are sources of selection on Monterey pine morphological traits; however, the nature and direction of that selection is driven by the likely specificity of the fungi involved.

The OTU *R. californiensis* had a particularly important role in selection; however, the strength and nature of that selection depended on plant fitness proxy. Selection was negative when the proportion survived was used as a plant fitness proxy but positive when total biomass was used as a plant fitness proxy. This difference in selection patterns based on fitness proxy suggests selection in plants for increased compatibility with *R. californiensis* would increase seedling biomass but decrease seedling survival. While we failed to identify a signal of significant selection when RGR was used as a fitness proxy, analysis of the G-matrix indicated positive genetic correlations of *R. californiensis* abundance with RGR, suggesting the potential for selection between the two traits*.* While not widely studied, *R. californiensis* was first identified in Monterey pine and California live oak forests, and public collection records (e.g., mycoportal.org and mushroomobserver.org) are largely restricted to coastal California, suggesting at least the potential of host specificity for this species^44^; more research is needed to understand whether this apparent specificity is genetically based or simply represents range restriction.

In contrast, we identified several generalist ECM fungal taxa that drove selection in the same way regardless of fitness proxy. Specifically, the abundances of two OTUs, *Tomentella1* and *T. sublilacina,* and their associated family, Thelephoraceae, experienced positive selection regardless of plant fitness proxy. Despite the consistent nature of fungi from the Thelephoraceae family to demonstrate patterns of natural selection with plant traits, G-matrix analysis only identified fungi from the OTU Thelephoraceae3 as experiencing significant genetic correlations with plant traits (negative with both biomass and RGR) while the other six OTUs from this family in the study failed to demonstrate significant genetic correlations with plant traits. Thus, apparent positive selection of ECM fungi in the Thelephoraceae on plant traits can best be interpreted as interspecific selection by fungal traits on plant traits, rather than simply correlated evolution of multiple plant traits. Positive selection for fungi from the genus *Tomentella* is perhaps not surprising given that these fungi are widespread, dominant species in mature forest stands, sporulate in the soil organic horizon, and can establish from the spore bank shortly after disturbance^45–47^. These characteristics suggest that selection may favor plant compatibility with fungi from this genus because they can provide benefits for the plant under a variety of conditions. However, positive selection on plants for increased compatibility with these fungi during the extreme drought conditions of this experiment may also indicate that Monterey pine may adapt to extreme climatic conditions via evolution of increased association with *Tomentella* and other Thelephoraceae fungi. It is perhaps unsurprising that the most abundant OTU recovered from our seedlings, *Tomentella1,* was involved in mycorrhizal mediated selection, as the net selective pressure exerted by mycorrhizal fungi on a particular plant trait may be dominated by the numerically most abundant member of the community^48^.

Our results suggest that the specificity of fungi involved in plant-mycorrhizal interactions has the potential to drive natural selection in opposing ways; however, for many mycorrhizal fungi we lack an understanding regarding their fidelity^49^. This research further emphasizes the need to bridge this important knowledge gap.

### Selection on exploration types

Exploration types, which reflect the species-level morphological traits by which ECM fungi acquire and transport nutrients, provide an integrated assessment of fungal function and may provide insight into how guilds of ECM fungi are exerting selection pressures^25,31,34,35^. In this study, there was selection by fungi from the contact exploration type on four different plant traits, suggesting they play an outsized role in the selection process. Fungi from the contact exploration type are hydrophilic but their ranges seem to be restricted by mean annual precipitation^50^, suggesting they may be important in dry conditions for plants to acquire water. The range of Monterey pine is coastal, but the soils where Monterey pine exist are generally dry as the pine acquires a large portion of its water budget from the annual fogbank, particularly in the summer when rainfall is limited^51,52^; these conditions were amplified in our study, which took place during an extreme drought event^53^. Taken together, these pieces of evidence suggest that selection on Monterey pine in these conditions has come to favor associations of the pine with ECM fungi that may alleviate water stress.

We also demonstrated instances of genetic correlations of exploration types with plant traits without identifying significant contemporary selection. For example, no plant traits experienced significant selection due to fungi from the short exploration type in any of the natural selection models despite positive genetic correlations of fungi from this exploration type with RGR and negative correlations with diameter. This could be because fungi from the short exploration type have previously exerted correlational selection on plant traits, such that the fitness of certain combinations of traits represented peaks on the adaptive landscape under different historical environmental conditions^54^. Alternatively, these genomic covariances of ECM fungal exploration types with plant morphological and fitness traits may result from correlated selection from other unmeasured environmental variables on both the mycorrhizal traits of the plant and these other plant traits.

### Selection on overall ECM fungal diversity

In support of correlational selection as a driving factor in natural selection of plant-fungal relationships is our finding that fungal symbiont diversity itself was an important source of selection. Diversity indices represent a quantitative measure for how many different ECM fungal species are present on the root, and thus may capture the effect of multiple mycorrhizal species as selective agents on the plant, and/or the outcome of selection (both from the environment and through interactions with other biota) on plants for their compatibility with individual fungal species. Interestingly, no particular combination of diversity and plant traits maximized both plant biomass or plant survival, suggesting the potential for antagonistic selection between plant traits and fungal community diversity, and for growth-survival trade-offs in plants. These patterns may reflect the complex nature of biotic selection, particularly for interactions whose function can vary from mutualistic to parasitic depending on resource availability^55,56^.

### Selection under drought conditions

This experiment took place during the hottest and driest period on record in the state of California^53^ and thus it is likely that selection favored combinations of plant and fungal traits more suited for desert-like conditions, as found more often on the Mexican islands (Cedros and Guadalupe) compared to the California mainland populations of Monterey pine (Supplementary Fig. S1^20^). Indeed, we found a significant advantage in survival for seedlings with an island (Guadalupe or Cedros) genetic background compared to seedlings with a mainland background (Cambria, Monterey, Año Nuevo), suggesting maladaptation of mainland genotypes to extreme drought conditions. Specifically, the odds of mortality for seedlings with a pure island background or hybrid between an island and mainland pine were 0.70 and 0.69 times the odds of mortality compared to a pure mainland genetic background. This result suggests that selection, especially when survival was used as a plant fitness proxy, favored traits that promote survival in hotter, drier climates over the wetter, cooler climates historically present at Cambria. Understanding selection on mycorrhizal relationships in Monterey pine and other trees during such an extreme climatic event is particularly important as climate change models predict increases in temperature and decreases in precipitation in the future for this region^57^. Moreover, these results lend insight into the microevolutionary processes that may underlie recently identified macroevolutionary patterns of dependent evolution between drought adaptation and mycorrhizal strategies in land plants^58^.

One of the biggest advantages of genotypic selection analysis is that it allows for the correction of the role of the environment on traits and thus decreases the possibility that the covariance between the environment and the trait(s) of interest leads to false conclusions regarding whether selection is acting on that trait^59,60^. This is perhaps most important in our study due to the extreme drought environment experienced by the plants and fungi used in this study. However, because we used family means to correct for environmental bias, outcomes of selection identified here are more likely to reflect genetic correlations rather than phenotypic correlations determined by the environment^60^.

## Conclusion

In this study, we provide evidence for natural selection in the mycorrhizal symbiosis between ECM fungi and Monterey Pine during one of the most extreme drought events on record in California. These results contribute to the growing body of evidence quantifying selection in multispecies interactions, especially bolstering our understanding of how coevolutionary selection operates in multispecific mutualisms. In particular, we demonstrate selection on plants for altered compatibility with specific fungal OTUs and families, with the direction and nature of this selection reflective of the apparent specificity of the fungi involved. We further demonstrate selection for particular fungal traits associated with the ability of the fungi to explore and acquire nutrients from the soil and the potential for genetic correlations between plant traits and specific fungal OTUs and exploration types. In total, this research represents an important first step in understanding multispecies coevolution; however, in order to fully understand this phenomenon in mycorrhizal interactions, common gardens that measure selection need to be replicated in other populations, which would allow estimation of geographic mosaics of coevolutionary selection^4,13^ in these multispecific mutualisms.

### Supplementary Information

Supplementary Information 1.

Supplementary Information 2.

Supplementary Information 3.

Supplementary Information 4.

Supplementary Information 5.

Supplementary Information 6.

## Data Availability

Seedling information including family, genetic background, source, the number planted, raw number survived, and proportion of the total planted that survived are found in Supplementary Table S1. The fungal sequence data for this project have been submitted to the GenBank databases under the accession numbers MN364462–MN364644.
