# Supplementary material for: Interspecific selection in a diverse mycorrhizal symbiosis
Source: Sci Rep. 2024 May 27;14:12151. doi: 10.1038/s41598-024-62815-4 (PMC11130337; doi:10.1038/s41598-024-62815-4)
Supplement: Supplementary file 1 — Supplementary Information 1. [file 41598_2024_62815_MOESM1_ESM.pdf]

Supplementary Material A: Model structures used in selection analyses. For all models, 'Plant Traits' include root:shoot, diameter and specific root length (SRL). Relative growth rate (RGR), biomass, and survival were all considered plant traits when not used as a proxy for plant fitness. 'EMF_Abund' describes the abundance of root tips colonized by ectomycorrhizal fungal.

Model 1: Basic Model Structure

	**Fitness Proxy**	**Model**
**a**	Biomass	Plant Traits + EMF_Abund + Plant Traits : EMF_Abund
**b**	RGR	Plant Traits + EMF_Abund + Plant Traits : EMF_Abund
**c**	Survival	Plant Traits + EMF_Abund + Plant Traits : EMF_Abund

Model 2: Most abundant operational taxonomic units (OTUs) or fungal families model structure.

	**Fitness Proxy**	**Model**
**a**	Biomass	Plant Traits + OTU + Plant Traits : OTU
**b**	RGR	Plant Traits + OTU + Plant Traits : OTU
**c**	Survival	Plant Traits + OTU + Plant Traits : OTU
**e**	Biomass	Plant Traits + Fungal Family + Plant Traits : Fungal Family
**f**	RGR	Plant Traits + Fungal Family + Plant Traits : Fungal Family
**g**	Survival	Plant Traits + Fungal Family + Plant Traits : Fungal Family

Model 3: Operation taxonomic unit (OTU) and fungal family main effect model structure

	**Fitness Proxy**	**Model**
**a**	Biomass	OTU
**b**	RGR	OTU

**c**	Survival	OTU
**d**	Biomass	Fungal Family
**e**	RGR	Fungal Family
**f**	Survival	Fungal Family

Model 4: Fungal hyphal biomass ('FungBiomass') model structure

	**Fitness Proxy**	**Model**
**a**	Biomass	Plant Traits + FungBiomass + FungBiomass : Plant Traits
**b**	RGR	Plant Traits + FungBiomass + FungBiomass : Plant Traits
**c**	Survival	Plant Traits + FungBiomass + FungBiomass : Plant Traits

Model 5: Exploration type ('ExploreType') model structure

		**Fitness Proxy**	**Model**
**a**	**Plant Fitness**	Biomass	Plant Traits + ExploreType + ExploreType : Plant Traits
**b**		RGR	Plant Traits + ExploreType + ExploreType : Plant Traits
**c**		Survival	Plant Traits + ExploreType + ExploreType : Plant Traits

Model 6: Alpha diversity model structure. Four proxies for alpha diversity were assessed: 'Observed', 'Chao1', 'Shannon' or 'Simpson'.

		**Fitness Proxy**	**Model**
**a**	**Plant Fitness**	Biomass	Plant Traits + Observed + Plant Traits : Observed
**b**		RGR	Plant Traits + Observed + Plant Traits : Observed
**c**		Survival	Plant Traits + Observed + Plant Traits : Observed
**d**		Biomass	Plant Traits + Chao1 + Plant Traits : Chao1

**e**		RGR	Plant Traits + Chao1 + Plant Traits : Chao1
**f**		Survival	Plant Traits + Chao1 + Plant Traits : Chao1
**g**		Biomass	Plant Traits + Shannon + Plant Traits : Shannon
**h**		RGR	Plant Traits + Shannon + Plant Traits : Shannon
**i**		Survival	Plant Traits + Shannon + Plant Traits : Shannon
**j**		Biomass	Plant Traits + Simpson + Plant Traits : Simpson
**k**		RGR	Plant Traits + Simpson + Plant Traits : Simpson
**l**		Survival	Plant Traits +Simpson + Plant Traits : Simpson

Supplementary Material B

Table S1. Seedling information including family, genetic background, source, the number planted, raw number survived, proportion of the total planted that survived, mean diameter and standard error at planting, and mean height and standard error at planting.

Family	Genetic Background	Source	Planted	Survived	Proportion Survived	Diameter (mm)	Height (cm)
Ano1	Año Nuevo	Mexus	30	14	0.47	1.07±0.04	11.1±0.25
Ano3	Año Nuevo	Mexus	30	14	0.47	1.09±0.05	7.60±0.30
Ano4	Año Nuevo	Mexus	30	13	0.43	1.01±0.05	7.01±0.27
Ano5	Año Nuevo	Mexus	30	24	0.80	1.08±0.03	8.83±0.26
Ano8	Año Nuevo	Mexus	30	8	0.27	0.94±0.03	8.82±0.32
Cam2	Cambria	Mexus	30	10	0.33	1.22±0.07	8.90±0.40
Cam3	Cambria	Mexus	30	10	0.33	1.25±0.06	10.6±0.34
Cam6	Cambria	Mexus	30	20	0.67	1.15±0.07	9.94±0.34
Cam9	Cambria	Mexus	30	14	0.47	1.15±0.04	11.9±0.39
Cam10	Cambria	Mexus	30	14	0.47	1.11±0.04	11.0±0.28
CedN14	Cedros North	Mexus	10	4	0.40	0.90±0.05	5.86±0.52
CedN19	Cedros North	Mexus	10	3	0.30	0.92±0.08	4.58±0.49
CedN24	Cedros North	Mexus	16	1	0.06	0.85±0.11	5.13±0.49
CedS6	Cedros South	Mexus	30	11	0.37	0.96±0.04	8.30±0.33
CedS7	Cedros South	Mexus	30	20	0.67	1.04±0.05	9.81±0.42
CedS9	Cedros South	Mexus	30	20	0.67	0.94±0.04	7.69±0.32
Guad4	Guadalupe	Mexus	5	1	0.20	1.00±0.12	7.64±0.61
Guad6	Guadalupe	Mexus	6	1	0.11	1.10±0.14	6.48±0.72
Guad7	Guadalupe	Mexus	15	0	0	0.88±0.06	5.43±0.35
Guad10	Guadalupe	Mexus	16	2	0.13	1.02±0.05	8.13±0.73
L3	Guadalupe	Russel Reserve	28	19	0.68	0.96±0.05	8.52±0.44
T6	Cedros	Russel Reserve	30	5	0.17	0.92±0.05	6.90±0.35
T8	Cedros	Russel Reserve	30	10	0.33	1.02±0.04	9.22±0.46
M10	Monterrey	Russel Reserve	8	0	0	1.08±0.13	8.86±1.25

M11	Monterrey	Russel Reserve	30	16	0.53	1.03±0.05	8.76±0.37
LxC17	Guadalupe x Cambria	Russel Reserve	30	12	0.40	1.09±0.04	8.70±0.38
LxC18	Guadalupe x Cambria	Russel Reserve	30	14	0.47	1.28±0.08	11.4±0.51
TxC20	Cedros x Cambria	Russel Reserve	15	2	0.13	0.88±0.06	7.42±0.58
TxC21	Cedros x Cambria	Russel Reserve	30	15	0.50	1.15±0.04	9.87±0.49
TxC22	Cedros x Cambria	Russel Reserve	30	17	0.57	1.21±0.04	4.53±3.42
TxC23	Cedros x Cambria	Russel Reserve	30	10	0.33	1.15±0.04	9.21±0.40
MxC24	Monterey x Cambria	Russel Reserve	30	13	0.43	1.25±0.06	10.6±0.43
LxT25	Guadalupe x Cedros	Russel Reserve	30	14	0.47	1.11±0.05	9.57±0.28
LxT26	Guadalupe x Cedros	Russel Reserve	30	9	0.30	0.97±0.06	7.95±0.53
TxL27	Cedros x Guadalupe	Russel Reserve	30	6	0.20	1.11±0.06	9.56±0.30
TxL28	Cedros x Guadalupe	Russel Reserve	30	16	0.53	1.19±0.05	10.5±0.37
LxM29	Guadalupe x Monterey	Russel Reserve	27	5	0.19	1.11±0.06	9.61±0.41
LxM30	Guadalupe x Monterey	Russel Reserve	18	1	0.06	0.92±0.06	6.84±0.52
MxT31	Monterey x Cedros	Russel Reserve	30	9	0.30	0.95±0.04	10.2±0.50
MxT32	Monterey x Cedros	Russel Reserve	30	13	0.43	1.19±0.05	10.3±0.43
MxT33	Monterey x Cedros	Russel Reserve	30	11	0.37	0.97±0.06	8.36±0.35
TxM34	Cedros x Monterey	Russel Reserve	30	9	0.30	1.03±0.05	9.79±0.48
MxTxA36	Monterey x Cedros x Año Nuevo	Russel Reserve	10	2	0.20	1.07±0.03	7.35±0.81
MxTxL37	Monterey x Cedros x Guadalupe	Russel Reserve	30	17	0.57	1.12±0.04	9.37±0.44
MxTxL38	Monterey x Cedros x Guadalupe	Russel Reserve	6	0	0	0.83±0.09	4.83±0.55
MxTxL39	Monterey x Cedros x Guadalupe	Russel Reserve	30	15	0.50	1.20±0.03	11.3±0.32
TxC40	Cedros x Cambria	Russel Reserve	25	8	0.32	1.13±0.07	10.6±0.43
**TOTAL / Average**			**1178**	**472**	**0.40**	**1.07±0.01**	**10.0±0.88**

Table S2. Phenotypic correlations among a) plant traits, b) fungal traits, and c) measures of alpha diversity.

a) Phenotypic correlations among plant traits

Trait	R : S	RGR (mm/day)	Diameter (mm)	SRL	Biomass (mg)	Proportion Survived
Root : Shoot	1	-0.2 ***	0.04	0.1	-0.17 **	0.14 *
RGR		1	0.12 *	-0.28 ***	0.65 ***	-0.02
Diameter			1	-0.08	0.17 **	0.03
SRL				1	-0.31 ***	-0.02
Biomass					1	-0.04
Proportion Survived						1

R : S = Root (mg) : Shoot (mg); RGR = relative growth rate; SRL = specific root length

b) Phenotypic correlations among fungal traits

Trait		Fungal Hyphal Biomass	Exploration Type					
			Contact	Long Distance	Medium Distance Fringe	Medium Distance Mat	Medium Distance Smooth	Short Distance
	Fungal Hyphal Biomass	1	-0.08	0.96 ***	0.11 *	0.1	-0.17 **	-0.23 ***
Exploration Type	Contact		1	-0.11 *	0.21 ***	-0.05	-0.03	-0.06
	Long Distance			1	-0.03	-0.06	-0.2 ***	-0.19 ***
	Medium Distance Fringe				1	-0.04	0.07	-0.15 **
	Medium Distance Mat					1	0.16 **	-0.2 ***

	Medium Distance Smooth						1	-0.86 ***
	Short Distance							1

c) Phenotypic correlations among measures of alpha diversity				
Trait	Shannon Index	Observed	Chao1	Simpson Index
Shannon Index	1	0.4 ***	0.38 ***	0.98 ***
Observed		1	1 ***	0.28 ***
Chao1			1	0.27 ***
Simpson Index				1

***, P < 0.001; **, P < 0.01; *, P < 0.05.

Table S3. Results (estimate ± standard error) of selection analysis for (a) total selection (differentials: S) and directional selection (selection gradients: β) for plant traits, fungal traits, and their interaction in fitness models for fungal hyphal biomass models (Supplementary Material A: Model 4). RGR = relative growth rate; SRL = specific root length

		Proportion Survived			*Biomass*			*RGR*		
**Trait**	**Source of Selection**	**Estimate**	**P-value**		**Estimate**	**P-value**		**Estimate**	**P-value**	
Root:Shoot	*S*	0.0836 ± 0.03	0.0030	**	-0.0878 ± 0.03	0.0022	**	-0.0241 ± 0.01	0.0003	***
	β	0.0834 ± 0.03	0.0024	**	-0.0191 ± 0.02	0.3857		-0.0109 ± 0.01	0.0344	*
Diameter	*S*	0.0341 ± 0.03	0.2099		0.0936 ± 0.03	0.0011	**	0.0152 ± 0.01	0.0230	*
	β	0.0250 ± 0.03	0.2618		0.0517 ± 0.02	0.0177	*	0.0010 ± 0.01	0.8485	
SRL	*S*	-0.0442 ± 0.03	0.1050		-0.1654 ± 0.03	<0.0001	***	-0.0341 ± 0.01	<0.0001	***
	β	-0.0494 ± 0.03	0.0922	.	-0.0692 ± 0.02	0.0022	**	-0.0097 ± 0.01	0.0669	.
RGR	*S*	0.0057 ± 0.03	0.8343		0.3472 ± 0.02	<0.0001	***	0.0806 ± 0.01	<0.0001	***
	β	0.0045 ± 0.03	0.9504		0.3214 ± 0.02	<0.0001	***	0.0754 ± 0.01	<0.0001	***
Fungal Hyphal Biomass	*S*	-0.0413 ± 0.03	0.1400		0.0330 ± 0.03	0.2520		0.0075 ± 0.01	0.2693	
	β	0.0076 ± 0.04	0.6799		0.0148 ± 0.03	0.6152		0.0050 ± 0.01	0.4940	
x Root:Shoot	*S*	0.0623 ± 0.03	0.0322	*	-0.0089 ± 0.03	0.7530		-0.0003 ± 0.01	0.9650	
	β	0.0535 ± 0.04	0.0710	.	-0.0019 ± 0.03	0.9530		0.0096 ± 0.01	0.2099	
x Diameter	*S*	0.0357 ± 0.03	0.2410		-0.0122 ± 0.03	0.7030		-0.0094 ± 0.01	0.2050	
	β	0.0110 ± 0.03	0.3667		0.0260 ± 0.03	0.3483		-0.0139 ± 0.01	0.0310	*
x SRL	*S*	0.0207 ± 0.03	0.5137		0.0300 ± 0.03	0.3720		-0.0017 ± 0.01	0.8320	
	β	0.0211 ± 0.03	0.6312		0.0474 ± 0.03	0.0821	.	-0.0086 ± 0.01	0.1520	
x RGR	*S*	-0.0401 ± 0.04	0.2716		-0.0550 ± 0.04	0.1500		-0.0190 ± 0.01	0.1830	
	β	-0.0129 ± 0.04	0.4967		0.0230 ± 0.03	0.4645		-0.0063 ± 0.01	0.3426	

Table S4. Results (estimate ± standard error) of selection analysis for (a) total selection (differentials: *S*) and directional selection (selection gradients: *β*) for plant traits, fungal traits, and their interaction in fitness models including exploration type (Supplementary Material A: Model 5). RGR = relative growth rate; SRL = specific root length

		Proportion Survived			*Biomass*			*RGR*		
**Trait**	**Source of Selection**	**Estimate**	**P-value**		**Estimate**	**P-value**		**Estimate**	**P-value**	
Root:Shoot	*S*	0.0836 ± 0.03	0.0030	**	-0.0878 ± 0.03	0.0022	**	-0.0241 ± 0.01	0.0003	***
	β	-0.0838 ± 0.03	0.9904		-0.0191 ± 0.02	0.3857		-0.0109 ± 0.01	0.0344	*
Diameter	*S*	0.0341 ± 0.03	0.2099		0.0936 ± 0.03	0.0011	**	0.0152 ± 0.01	0.0230	*
	β	-0.2539 ± 0.07	0.9977		0.0517 ± 0.02	0.0177	*	0.0010 ± 0.01	0.8485	
SRL	*S*	-0.0442 ± 0.03	0.1050		-0.1654 ± 0.03	<0.0001	***	-0.0341 ± 0.01	<0.0001	***
	β	0.1217 ± 0.03	0.9962		-0.0692 ± 0.02	0.0022	**	-0.0097 ± 0.01	0.0669	.
RGR	*S*	0.0057 ± 0.03	0.8343		0.3472 ± 0.02	<0.0001	***	0.0806 ± 0.01	<0.0001	***
	β	-0.4874 ± 0.15	0.9993		0.3214 ± 0.02	<0.0001	***	0.0754 ± 0.01	<0.0001	***
Contact	*S*	0.0360 ± 0.03	0.1868		-0.0529 ± 0.03	0.0661	.	-0.0098 ± 0.01	0.1430	
	β	-0.5565 ± 0.15	0.0284	*	-0.0241 ± 0.03	0.3908		0.0013 ± 0.01	0.8457	
x Root:Shoot	*S*	0.0470 ± 0.02	0.0762		-0.0400 ± 0.03	0.1320		-0.0131 ± 0.01	0.0329	*
	β	-0.0316 ± 0.05	0.4959		0.0163 ± 0.04	0.6819		-0.0231 ± 0.01	0.0153	*
x Diameter	*S*	-0.0067 ± 0.02	0.7219		-0.0478 ± 0.02	0.0164	*	-0.0044 ± 0.01	0.3470	
	β	-0.0359 ± 0.02	0.1261		-0.0856 ± 0.02	<0.0001	***	0.0055 ± 0.01	0.2930	
x SRL	*S*	0.0717 ± 0.03	0.0216	*	0.0002 ± 0.03	0.9940		-0.0092 ± 0.01	0.2110	
	β	-0.0314 ± 0.04	0.4943		0.0467 ± 0.03	0.1745		-0.0048 ± 0.01	0.7522	
x RGR	*S*	-0.0465 ± 0.03	0.1503		-0.0705 ± 0.03	0.0372	*	-0.0173 ± 0.01	0.0319	*
	β	-0.0769 ± 0.04	0.0420	*	-0.0139 ± 0.03	0.6854		-0.0028 ± 0.01	0.7522	
Long Distance	*S*	-0.0491 ± 0.03	0.0790		0.0548 ± 0.03	0.0569	.	0.0114 ± 0.01	0.0894	.
	β	0.0659 ± 0.03	0.0028	**	0.0039 ± 0.06	0.9477		0.0204 ± 0.01	0.1496	
x Root:Shoot	*S*	0.0486 ± 0.03	0.0973		-0.0030 ± 0.03	0.9200		0.0028 ± 0.01	0.6830	
	β	-0.1307 ± 0.10	0.4111		0.1265 ± 0.08	0.1277		-0.0378 ± 0.02	0.0576	.
x Diameter	*S*	0.0362 ± 0.03	0.2128		0.0060 ± 0.03	0.8440		-0.0061 ± 0.01	0.3890	
	β	-0.0392 ± 0.05	0.4333		-0.0910 ± 0.05	0.0466	*	0.0086 ± 0.01	0.4680	
x SRL	*S*	-0.0082 ± 0.03	0.7750		0.0237 ± 0.03	0.4360		-0.0011 ± 0.01	0.8720	
	β	-0.1010 ± 0.08	0.1545		0.1321 ± 0.07	0.0467	*	-0.0061 ± 0.02	0.7100	
x RGR	*S*	-0.0403 ± 0.03	0.2413		-0.0181 ± 0.04	0.6140		-0.0039 ± 0.01	0.6270	
	β	-0.1441 ± 0.07	0.0231	*	0.1577 ± 0.06	0.0063	**	-0.0047 ± 0.01	0.1780	
Medium Distance Fringe	*S*	0.1650 ± 0.03	0.9690		0.0256 ± 0.03	0.3750		-0.0051 ± 0.01	0.4500	
	β	0.0212 ± 0.03	0.9746		0.0142 ± 0.03	0.5814		0.0062 ± 0.01	0.3140	

x Root:Shoot	*S*	0.0415 ± 0.02	0.0300	*	-0.0267 ± 0.02	0.1270		-0.0069 ± 0.01	0.0882	.
	β	-0.0373 ± 0.03	0.9985		0.0096 ± 0.02	0.6674		-0.0074 ± 0.01	0.1780	
x Diameter	*S*	0.0008 ± 0.02	0.9731		-0.0444 ± 0.03	0.0835	.	-0.0101 ± 0.01	0.0891	.
	β	-0.0180 ± 0.03	0.9990		-0.0400 ± 0.02	0.0942	.	0.0011 ± 0.01	0.8584	
x SRL	*S*	0.0579 ± 0.03	0.0337	*	-0.0299 ± 0.03	0.2700		-0.0113 ± 0.01	0.0711	.
	β	0.0074 ± 0.04	0.9986		-0.0101 ± 0.03	0.7399		-0.0055 ± 0.01	0.5299	
x RGR	*S*	-0.0227 ± 0.03	0.3891		-0.0331 ± 0.03	0.2340		-0.0133 ± 0.01	0.1170	
	β	-0.0150 ± 0.03	0.9994		-0.0337 ± 0.03	0.1907		-0.0055 ± 0.01	0.5299	
Medium Distance Smooth	*S*	0.0423 ± 0.03	0.1200		-0.0625 ± 0.03	0.0298	*	-0.0104 ± 0.01	0.1190	
	β	-0.0559 ± 0.03	0.0137	*	-0.0396 ± 0.12	0.7451		0.0163 ± 0.03	0.5732	
x Root:Shoot	*S*	-0.0691 ± 0.03	0.0299	*	0.0200 ± 0.03	0.5430		-0.0119 ± 0.01	0.1200	
	β	-0.3428 ± 0.20	0.1244		0.2929 ± 0.17	0.0852	.	-0.1280 ± 0.04	0.0017	**
x Diameter	*S*	0.0237 ± 0.03	0.4377		0.0511 ± 0.03	0.1150		0.0078 ± 0.01	0.3030	
	β	-0.0860 ± 0.10	0.2949		-0.2235 ± 0.08	0.0081	**	0.0525 ± 0.02	0.0218	*
x SRL	*S*	-0.0131 ± 0.02	0.5830		-0.0039 ± 0.03	0.8770		0.0058 ± 0.01	0.3210	
	β	-0.1936 ± 0.17	0.3307		0.1918 ± 0.14	0.1850		0.0025 ± 0.04	0.9436	
x RGR	*S*	0.0352 ± 0.02	0.1422		-0.0530 ± 0.02	0.0336	*	-0.0152 ± 0.01	0.0180	*
	β	-0.2489 ± 0.14	0.0893	.	0.2772 ± 0.12	0.0207	*	-0.0118 ± 0.03	0.7272	
Short Distance	*S*	-0.0400 ± 0.03	0.1403		0.0500 ± 0.03	0.0824	.	0.0091 ± 0.01	0.1750	
	β	0.0030 ± 0.03	0.0048	**	-0.0240 ± 0.13	0.8489		0.0205 ± 0.03	0.4947	
x Root:Shoot	*S*	0.0112 ± 0.03	0.7223		-0.0173 ± 0.03	0.6070		0.0093 ± 0.01	0.2320	
	β	-0.2623 ± 0.21	0.2485		0.2814 ± 0.18	0.1102		-0.1159 ± 0.04	0.0061	**
x Diameter	*S*	-0.0545 ± 0.03	0.0553	.	-0.0362 ± 0.03	0.2260		-0.0023 ± 0.01	0.7440	
	β	-0.1674 ± 0.11	0.1267		-0.2780 ± 0.09	0.0019	**	0.0491 ± 0.02	0.0469	*
x SRL	*S*	0.0044 ± 0.03	0.8616		-0.0056 ± 0.03	0.8350		-0.0051 ± 0.01	0.4090	
	β	-0.2294 ± 0.18	0.2453		0.1987 ± 0.15	0.1904		0.0027 ± 0.04	0.9418	
x RGR	*S*	-0.0346 ± 0.02	0.1539		0.0720 ± 0.03	0.0045	**	0.0191 ± 0.01	0.0024	**
	β	-0.3052 ± 0.15	0.0458	*	0.3246 ± 0.13	0.0112	*	-0.0009 ± 0.03	0.9789	

***, $P < 0.001$; **, $P < 0.01$; *, $P < 0.05$.

Table S5. Results (estimate ± standard error) for total selection (differentials: *S*) and directional selection (selection gradients: *β*) for fungal OTUs and their families (bold) in fitness models with no interactions (Supplementary Material A: Model 3).

		Proportion Survived			*Biomass*			*RGR*		
**OTU/Family**	**Source of Selection**	**Estimate**	**P-value**		**Estimate**	**P-value**		**Estimate**	**P-value**	
**Atheliaceae**	*S*	0.0514 ± 0.03	0.2080		0.0550 ± 0.03	0.0622	.	-0.0005 ± 0.01	0.9490	
	β	0.0497 ± 0.03	0.2260		0.0853 ± 0.03	0.0015	**	0.0560 ± 0.01	0.4440	
Atheliaceae1	*S*	0.0396 ± 0.03	0.4870		0.0703 ± 0.03	0.0132	*	0.0030 ± 0.01	0.7050	
	β	0.0073 ± 0.04	0.5940		0.0699 ± 0.03	0.0414	*	0.0077 ± 0.01	0.4112	
Atheliaceae2	*S*	0.0603 ± 0.03	0.7240		0.0500 ± 0.03	0.0901	.	0.0004 ± 0.01	0.9600	
	β	0.0525 ± 0.04	0.2960		0.0280 ± 0.03	0.4136		-0.0007 ± 0.01	0.9392	
Tylospora1	*S*	-0.0118 ± 0.03	0.7240		-0.0521 ± 0.03	0.0777	.	-0.0100 ± 0.01	0.2010	
	β	-0.0109 ± 0.03	0.7490		-0.0187 ± 0.03	0.5018		-0.0002 ± 0.01	0.9790	
**Gloniaceae**	*S*	-0.0160 ± 0.03	0.6320		0.0215 ± 0.03	0.4680		0.0067 ± 0.01	0.3960	
	β	-0.0078 ± 0.03	0.8370		0.0731 ± 0.03	0.0081	**	0.0069 ± 0.01	0.3558	
Cenococcum	*S*	-0.0160 ± 0.03	0.6320		0.2150 ± 0.03	0.4680		0.0067 ± 0.01	0.3960	
	β	-0.0023 ± 0.04	0.9500		0.0641 ± 0.03	0.0257	*	0.0105 ± 0.01	0.1921	
**Pezizaceace**	*S*	-0.0264 ± 0.03	0.5210		0.0873 ± 0.03	0.0030	**	0.0119 ± 0.01	0.1310	
	β	-0.0256 ± 0.03	0.5330		0.0958 ± 0.03	0.0003	***	0.0148 ± 0.01	0.0446	*
Pezizaceae1	*S*	-0.0264 ± 0.03	0.5210		0.0873 ± 0.03	0.0030	**	0.0119 ± 0.01	0.1310	
	β	-0.0241 ± 0.03	0.5380		0.0942 ± 0.03	0.0007	***	0.0150 ± 0.01	0.0450	*
**Russulaceae**	*S*	-0.0230 ± 0.03	0.4910		0.0295 ± 0.03	0.3190		0.0166 ± 0.01	0.0340	*
	β	-0.0186 ± 0.04	0.6270		0.0794 ± 0.03	0.0037	**	0.0258 ± 0.01	0.0009	***
Russula1	*S*	-0.0429 ± 0.03	0.3450		-0.0159 ± 0.03	0.5910		-0.0056 ± 0.01	0.4780	
	β	-0.0355 ± 0.03	0.3400		-0.0007 ± 0.03	0.9810		-0.0027 ± 0.01	0.7296	
Russula2	*S*	0.0425 ± 0.03	0.2890		0.0348 ± 0.03	0.2390		0.0035 ± 0.01	0.6540	
	β	0.0415 ± 0.03	0.2920		0.0394 ± 0.03	0.1590		0.0054 ± 0.01	0.4746	
Russula californiensis	*S*	-0.0330 ± 0.03	0.3300		0.0399 ± 0.03	0.1770		0.0178 ± 0.01	0.0226	*
	β	-0.0144 ± 0.04	0.7330		0.0773 ± 0.03	0.0079	**	0.0230 ± 0.01	0.0047	**
Russula xerampelina	*S*	0.0190 ± 0.03	0.5820		0.0133 ± 0.03	0.6540		0.0069 ± 0.01	0.3810	
	β	0.0196 ± 0.03	0.5780		0.0281 ± 0.03	0.3044		0.0010 ± 0.01	0.1854	
Russulaceae1	*S*	-0.0330 ± 0.03	0.2180		-0.0271 ± 0.03	0.3660		0.0042 ± 0.01	0.5930	
	β	-0.0358 ± 0.04	0.3260		-0.0194 ± 0.03	0.4985		0.0065 ± 0.01	0.4088	
**Sebacinaceae**	*S*	-0.0136 ± 0.03	0.6850		0.0210 ± 0.03	0.4770		-0.0062 ± 0.01	0.4290	
	β	-0.0133 ± 0.03	0.7160		0.0536 ± 0.03	0.0454	*	0.0012 ± 0.01	0.8717	
Sebacinaceae1	*S*	-0.0136 ± 0.03	0.6850		0.0210 ± 0.03	0.4770		-0.0062 ± 0.01	0.4290	
	β	-0.0126 ± 0.03	0.7090		0.0483 ± 0.03	0.0816	.	0.0020 ± 0.01	0.7975	
**Thelephoraceae**	*S*	0.0011 ± 0.03	0.9740		0.1483 ± 0.03	<0.0001	***	0.0364 ± 0.01	<0.0001	***
	β	-0.0002 ± 0.04	0.9710		0.2043 ± 0.03	<0.0001	***	0.0444 ± 0.01	<0.0001	***
Thelephoraceae1	*S*	0.0580 ± 0.03	0.0950	.	0.0230 ± 0.03	0.4360		0.0043 ± 0.01	0.5840	
	β	0.0448 ± 0.04	0.2420		0.0562 ± 0.03	0.0496	*	0.0164 ± 0.01	0.0394	*
Thelephoraceae2	*S*	-0.0289 ± 0.03	0.5560		0.0144 ± 0.03	0.6270		0.0066 ± 0.01	0.4020	
	β	-0.0285 ± 0.03	0.5580		0.0262 ± 0.03	0.3380		0.0103 ± 0.01	0.1667	
Thelephoraceae5	*S*	-0.0248 ± 0.03	0.4740		0.0479 ± 0.03	0.1050		0.0060 ± 0.01	0.4460	
	β	-0.0245 ± 0.03	0.4840		0.0608 ± 0.03	0.0280	*	0.0104 ± 0.01	0.1679	
Tomentella1	*S*	0.0097 ± 0.03	0.7720		0.0620 ± 0.03	0.0354	*	0.0154 ± 0.01	0.0492	*
	β	0.0088 ± 0.03	0.8130		0.0876 ± 0.03	0.0017	**	0.0230 ± 0.01	0.0027	**
Tomentella4	*S*	0.0229 ± 0.03	0.5050		0.0347 ± 0.03	0.2410		0.0098 ± 0.01	0.2120	
	β	0.0206 ± 0.03	0.8580		0.0316 ± 0.03	0.2493		0.0098 ± 0.01	0.1920	
Tomentella sublilacina	*S*	0.0106 ± 0.03	0.7490		0.0978 ± 0.03	<0.0001	***	0.0243 ± 0.01	0.0018	**
	β	0.0070 ± 0.04	0.5500		0.1471 ± 0.03	<0.0001	***	0.0382 ± 0.01	<0.0001	***

**Tuberaceae**	*S*	0.0273 ± 0.03	0.4390		0.0287 ± 0.03	0.3320		-0.0047 ± 0.01	0.5490	
	β	0.0263 ± 0.03	0.4520		0.0517 ± 0.03	0.0525	.	0.0014 ± 0.01	0.8535	
Tuber1	*S*	0.0273 ± 0.03	0.4390		0.0287 ± 0.03	0.3320		-0.0047 ± 0.01	0.5490	
	β	0.0278 ± 0.03	0.4390		0.0492 ± 0.03	0.0741	.	0.0018 ± 0.01	0.8148	
**Unclassified**	*S*	0.0062 ± 0.03	0.8510		0.0683 ± 0.03	0.0203	*	0.0090 ± 0.01	0.2540	
	β	0.0075 ± 0.03	0.8080		0.0979 ± 0.03	0.0003	***	0.0105 ± 0.01	0.1531	
Helotiales2	*S*	-0.0465 ± 0.03	0.2910		0.0288 ± 0.03	0.0030	**	0.0119 ± 0.01	0.1310	
	β	-0.0444 ± 0.03	0.3290		0.0942 ± 0.02	0.0007	***	0.0150 ± 0.01	0.0450	*

***, P < 0.001; **, P < 0.01; *, P < 0.05.

Table S6. Results (estimate ± standard error) of selection analysis for total selection (differentials: *S*) and directional selection (selection gradients: *β*) for plant traits, measurements of alpha diversity, and their interaction in fitness models and on the plant trait resulting from its covariance with the measurements of alpha diversity (Supplementary Material A: Model 6). RGR = relative growth rate; SRL = specific root length

		Proportion Survived			*Biomass*			*RGR*		
**Trait**	**Source of Selection**	**Estimate**	**P-value**		**Estimate**	**P-value**		**Estimate**	**P-value**	
Observed										
Root:Shoot	*S*	0.0680 ± 0.03	0.0373	*	-0.0884 ± 0.03	0.0020	**	-0.0278 ± 0.01	0.0003	***
	β	0.0903 ± 0.03	0.0070	**	-0.0453 ± 0.02	0.0355	*	-0.0125 ± 0.01	0.0446	*
Diameter	*S*	-0.0004 ± 0.03	0.9900		0.0918 ± 0.03	0.0014	**	0.0103 ± 0.01	0.1870	*
	β	0.0058 ± 0.03	0.8243		0.0384 ± 0.02	0.0667	.	0.0019 ± 0.01	0.7498	
SRL	*S*	-0.0565 ± 0.03	0.0778	.	-0.1656 ± 0.03	<0.0001	***	-0.0354 ± 0.01	<0.0001	***
	β	-0.0549 ± 0.03	0.1018		-0.0863 ± 0.02	<0.0001	***	-0.0103 ± 0.01	0.1088	
RGR	*S*	0.0120 ± 0.03	0.7050		0.3458 ± 0.02	<0.0001	***	0.0788 ± 0.01	<0.0001	***
	β	0.0037 ± 0.03	0.9298		0.3072 ± 0.02	<0.0001	***	0.0726 ± 0.01	<0.0001	***
Observed	*S*	-0.0347 ± 0.03	0.2750		0.1415 ± 0.03	<0.0001	***	0.0182 ± 0.01	0.0198	*
	β	-0.0504 ± 0.03	0.1393		0.1027 ± 0.02	<0.0001	***	-0.0016 ± 0.01	0.7980	
x Root:Shoot	*S*	0.0341 ± 0.03	0.2990		0.0370 ± 0.03	0.1950		0.0163 ± 0.01	0.0400	*
	β	0.0491 ± 0.03	0.1485		-0.0002 ± 0.02	0.9911		0.0115 ± 0.01	0.0677	.
x Diameter	*S*	0.0655 ± 0.03	0.0416	*	0.0298 ± 0.03	0.2820		0.0102 ± 0.01	0.1840	
	β	0.0725 ± 0.03	0.0271	*	-0.0002 ± 0.02	0.9911		0.0061 ± 0.01	0.3056	
x SRL	*S*	0.0449 ± 0.03	0.1810		0.0271 ± 0.03	0.3860		0.0122 ± 0.01	0.1350	
	β	0.0443 ± 0.03	0.2013		0.0063 ± 0.02	0.7874		0.0089 ± 0.01	0.1707	
x RGR	*S*	-0.0418 ± 0.04	0.2400		0.0273 ± 0.03	0.3970		0.0070 ± 0.01	0.3770	
	β	-0.0444 ± 0.04	0.2252		0.0782 ± 0.02	0.0012	**	-0.0029 ± 0.01	0.6514	
Chao1										
Root:Shoot	*S*	0.0680 ± 0.03	0.0373	*	-0.0884 ± 0.03	0.0020	**	-0.0278 ± 0.01	0.0003	***
	β	0.0903 ± 0.03	0.0070	**	-0.0450 ± 0.02	0.0370	*	-0.0124 ± 0.01	0.0460	*
Diameter	*S*	-0.0004 ± 0.03	0.9900		0.0918 ± 0.03	0.0014	**	0.0103 ± 0.01	0.7489	
	β	0.0058 ± 0.03	0.8243		0.0373 ± 0.02	0.0745	.	0.0019 ± 0.01	0.8263	
SRL	*S*	-0.0565 ± 0.03	0.0778	.	-0.1656 ± 0.03	<0.0001	***	-0.0354 ± 0.01	<0.0001	***
	β	-0.0549 ± 0.03	0.1018		-0.0857 ± 0.02	<0.0001	***	-0.0102 ± 0.01	0.1170	
RGR	*S*	0.0120 ± 0.03	0.7050		0.3458 ± 0.02	<0.0001	***	0.0788 ± 0.01	<0.0001	***
	β	0.0037 ± 0.03	0.9298		0.3089 ± 0.02	<0.0001	***	0.0727 ± 0.01	<0.0001	***
Chao1	*S*	-0.0363 ± 0.03	0.2530		0.1366 ± 0.03	<0.0001	***	0.0175 ± 0.01	0.0247	*
	β	-0.0543 ± 0.03	0.1086		0.1010 ± 0.02	<0.0001	***	-0.0019 ± 0.01	0.7698	
x Root:Shoot	*S*	0.0349 ± 0.03	0.2950		0.0387 ± 0.03	0.1810		0.0168 ± 0.01	0.0374	*
	β	0.0520 ± 0.03	0.1296		-0.0322 ± 0.02	0.1387		0.0119 ± 0.01	0.0629	.
x Diameter	*S*	0.0614 ± 0.03	0.0544	.	0.0263 ± 0.03	0.3410		0.0095 ± 0.01	0.2130	
	β	0.0704 ± 0.03	0.0295	*	-0.0044 ± 0.02	0.8274		0.0060 ± 0.01	0.3110	
x SRL	*S*	0.0467 ± 0.03	0.1700		0.0928 ± 0.03	0.3470		0.0126 ± 0.01	0.1260	
	β	0.0469 ± 0.03	0.1799		0.0092 ± 0.02	0.6960		0.0092 ± 0.01	0.1617	
x RGR	*S*	-0.0403 ± 0.04	0.2610		0.0259 ± 0.03	0.4260		0.0068 ± 0.01	0.3940	
	β	-0.0421 ± 0.04	0.2524		0.0840 ± 0.02	0.0005	***	-0.0028 ± 0.01	0.6656	
Shannon Index										
Root:Shoot	*S*	0.0680 ± 0.03	0.0373	*	-0.0884 ± 0.03	0.0020	**	-0.0278 ± 0.01	0.0003	***
	β	0.0954 ± 0.03	0.0047	**	-0.0490 ± 0.02	0.0173	*	-0.0163 ± 0.01	0.0101	*
Diameter	*S*	-0.0004 ± 0.03	0.9900		0.0918 ± 0.03	0.0014	**	0.0103 ± 0.01	0.1870	*
	β	-0.0132 ± 0.03	0.7321		0.0225 ± 0.02	0.2946		0.0014 ± 0.01	0.8263	

SRL	*S*	-0.0565 ± 0.03	0.0778	.	-0.1656 ± 0.03	<0.0001	***	-0.0354 ± 0.01	<0.0001	***
	β	-0.0576 ± 0.03	0.0866	.	-0.0847 ± 0.02	<0.0001	***	-0.0101 ± 0.01	0.1230	.
RGR	*S*	0.0120 ± 0.03	0.7050		0.3458 ± 0.02	<0.0001	***	0.0788 ± 0.01	<0.0001	***
	β	0.0366 ± 0.04	0.3171		0.2738 ± 0.02	<0.0001	***	0.0728 ± 0.01	<0.0001	***
Shannon	*S*	-0.0447 ± 0.03	0.1610		0.2359 ± 0.03	<0.0001	***	-0.0332 ± 0.01	<0.0001	***
	β	-0.0702 ± 0.04	0.0666	.	0.1583 ± 0.02	<0.0001	***	0.0094 ± 0.01	0.2087	
x Root:Shoot	*S*	0.0347 ± 0.03	0.3200		0.0070 ± 0.03	0.8140		0.0034 ± 0.01	0.6860	
	β	0.0352 ± 0.03	0.2914		-0.0133 ± 0.02	0.5155		0.0042 ± 0.01	0.5165	
x Diameter	*S*	0.0278 ± 0.03	0.3640		0.0124 ± 0.03	0.6490		-0.0028 ± 0.01	0.7080	
	β	0.0629 ± 0.03	0.0553	.	-0.0203 ± 0.02	0.3307		-0.0075 ± 0.01	0.2416	
x SRL	*S*	0.0407 ± 0.04	0.3000		-0.0202 ± 0.04	0.5800		0.0042 ± 0.01	0.6610	
	β	-0.0109 ± 0.04	0.7819		0.0193 ± 0.03	0.4892		0.0051 ± 0.01	0.5272	
x RGR	*S*	-0.0509 ± 0.03	0.1320		0.0769 ± 0.03	0.0132	*	0.0145 ± 0.01	0.0358	*
	β	-0.0304 ± 0.04	0.4267		0.0737 ± 0.02	0.0016	**	-0.0100 ± 0.01	0.1011	
Simpson Index										
Root:Shoot	*S*	0.0680 ± 0.03	0.0373	*	-0.0884 ± 0.03	0.0020	**	-0.0278 ± 0.01	0.0003	***
	β	0.0903 ± 0.03	0.0070	**	-0.0428 ± 0.02	0.0413	*	-0.0165 ± 0.01	0.0092	**
Diameter	*S*	-0.0004 ± 0.03	0.9900		0.0918 ± 0.03	0.0014	**	0.0103 ± 0.01	0.1870	*
	β	0.0058 ± 0.03	0.8243		0.0287 ± 0.02	0.1968		0.0016 ± 0.01	0.8140	
SRL	*S*	-0.0565 ± 0.03	0.0778	.	-0.1656 ± 0.03	<0.0001	***	-0.0354 ± 0.01	<0.0001	***
	β	-0.0549 ± 0.03	0.1018		-0.0832 ± 0.02	<0.0001	***	-0.0102 ± 0.01	0.1186	
RGR	*S*	0.0120 ± 0.03	0.7050		0.3458 ± 0.02	<0.0001	***	0.0788 ± 0.01	<0.0001	***
	β	0.0037 ± 0.03	0.9298		0.2784 ± 0.02	<0.0001	***	0.0723 ± 0.01	<0.0001	***
Simpson	*S*	-0.0502 ± 0.03	0.1160		0.2253 ± 0.03	<0.0001	***	0.0324 ± 0.01	<0.0001	***
	β	-0.0688 ± 0.04	0.0683	.	0.1401 ± 0.02	<0.0001	***	0.0099 ± 0.01	0.1755	
x Root:Shoot	*S*	0.0362 ± 0.04	0.3230		0.0013 ± 0.03	0.9690		0.0018 ± 0.01	0.8410	
	β	0.0386 ± 0.04	0.2714		0.0004 ± 0.02	0.9863		0.0035 ± 0.01	0.6150	
x Diameter	*S*	0.0137 ± 0.03	0.6460		0.0056 ± 0.03	0.8360		-0.0033 ± 0.01	0.6570	
	β	0.0470 ± 0.03	0.1402		-0.0194 ± 0.02	0.3572		-0.0076 ± 0.01	0.2292	
x SRL	*S*	0.0406 ± 0.04	0.2830		-0.0228 ± 0.04	0.5190		0.0035 ± 0.01	0.7030	
	β	-0.0044 ± 0.04	0.9054		0.0043 ± 0.03	0.8729		0.0045 ± 0.01	0.5522	
x RGR	*S*	-0.0471 ± 0.03	0.1540		0.0740 ± 0.03	0.0141	*	0.0147 ± 0.01	0.0335	*
	β	-0.0219 ± 0.04	0.5852		0.0649 ± 0.02	0.0048	**	-0.0093 ± 0.01	0.1224	

***, P < 0.001; **, P < 0.01; *, P < 0.05.

Table S7. Results (estimate ± standard error) of selection analysis for (a) total selection (differentials: *S*) and directional selection (selection gradients: *β*) for plant traits and (b) on the plant trait resulting from its covariance with the fungal trait (Supplementary Material A: Model 1). RGR = relative growth rate; SRL = specific root length

		Proportion Survived			*Biomass*			*RGR*		
**Trait**	**Source of Selection**	**Estimate**	**P-value**		**Estimate**	**P-value**		**Estimate**	**P-value**	
Root:Shoot	*S*	0.0680 ± 0.03	0.0373	*	-0.0878 ± 0.03	0.0022	**	-0.0278 ± 0.01	0.0003	***
	β	0.0965 ± 0.04	0.0068	**	-0.0399 ± 0.02	0.0589	.	-0.0163 ± 0.01	0.0151	*
Diameter	*S*	-0.0004 ± 0.03	0.9900		0.0936 ± 0.03	0.0011	**	0.0103 ± 0.01	0.1870	
	β	-0.0019 ± 0.03	0.9603		0.0412 ± 0.02	0.0468	*	0.0029 ± 0.01	0.6252	
SRL	*S*	-0.0565 ± 0.03	0.0778	.	-0.1654 ± 0.03	<0.0001	***	-0.0354 ± 0.01	<0.0001	***
	β	-0.0570 ± 0.04	0.1143		-0.0540 ± 0.02	0.0177	*	-0.0094 ± 0.01	0.1544	
RGR/Biomass	*S*	0.0120 ± 0.03	0.7050		0.3472 ± 0.02	<0.0001	***	0.0788 ± 0.01	<0.0001	***
	β	0.0189 ± 0.04	0.7447		0.2722 ± 0.02	<0.0001	***	0.0667 ± 0.01	<0.0001	***
Tip Abundance	*S*	-0.0198 ± 0.03	0.5330		0.2270 ± 0.03	<0.0001	***	0.0522 ± 0.01	<0.0001	***
	β	-0.0303 ± 0.05	0.5376		0.1530 ± 0.03	<0.0001	***	0.0244 ± 0.01	0.0197	*
x Root:Shoot	*S*	0.0221 ± 0.04	0.5440		0.0111 ± 0.03	0.7330		0.0188 ± 0.01	0.0355	*
	β	0.0425 ± 0.04	0.2782	.	-0.0398 ± 0.02	0.1076		0.0054 ± 0.01	0.4588	
x Diameter	*S*	0.0029 ± 0.02	0.8940		0.0173 ± 0.02	0.3720		0.0040 ± 0.01	0.4470	
	β	0.0155 ± 0.02	0.3569		-0.0074 ± 0.01	0.6084		-0.0016 ± 0.004	0.7068	*
x SRL	*S*	0.0598 ± 0.04	0.1070		-0.0484 ± 0.03	0.1250		-0.0009 ± 0.01	0.9200	
	β	0.0166 ± 0.05	0.6601		0.0239 ± 0.03	0.4228	.	0.0030 ± 0.01	0.7377	
x RGR/Biomass	*S*	-0.0503 ± 0.04	0.0898	.	0.0209 ± 0.03	0.4610		0.0132 ± 0.01	0.0346	*
	β	-0.0355 ± 0.04	0.2910		-0.0055 ± 0.03	0.8446		-0.0190 ± 0.01	0.0028	**

***, P < 0.001; **, P < 0.01; *, P < 0.05.

Figure S1. Soil and climate data for each of the six native populations of *Pinus radiata*.

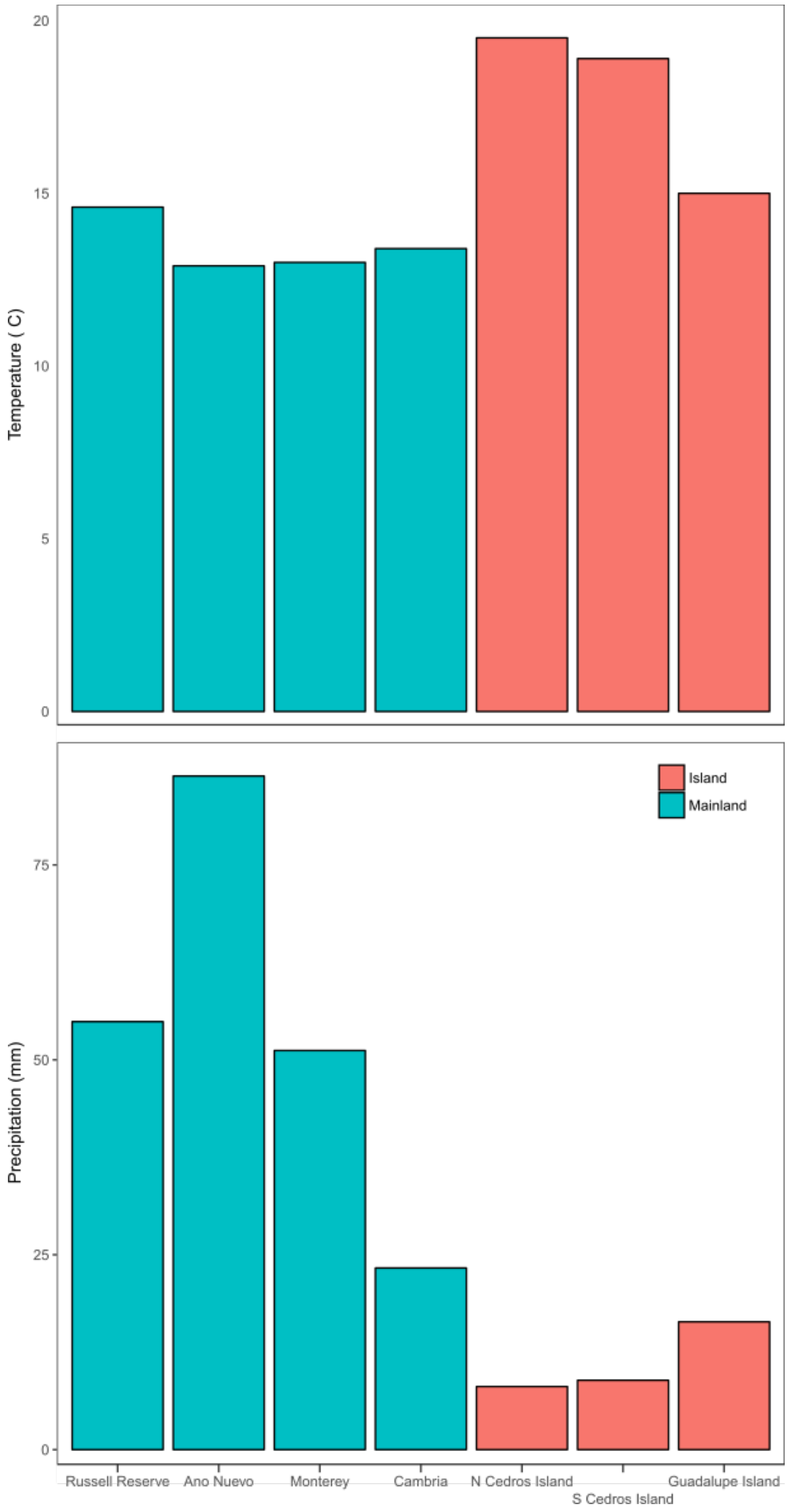

Figure S2. Relative abundances of major ectomycorrhizal fungal a) OTUs and b) exploration types recovered from seedlings, across Monterey pine genetic backgrounds planted in a common garden experiment. Relative abundance of OTUs/Exploration Types was calculated as the proportion of sequences belonging to a particular lineage of all ITS gene sequences recovered from each seedling. Sequences were pooled by seedling genetic background. Only those OTUs that represent >90% of the total tips are identified separately via color while the remaining OTUs were grouped into an 'Other' category for visualization.

a)

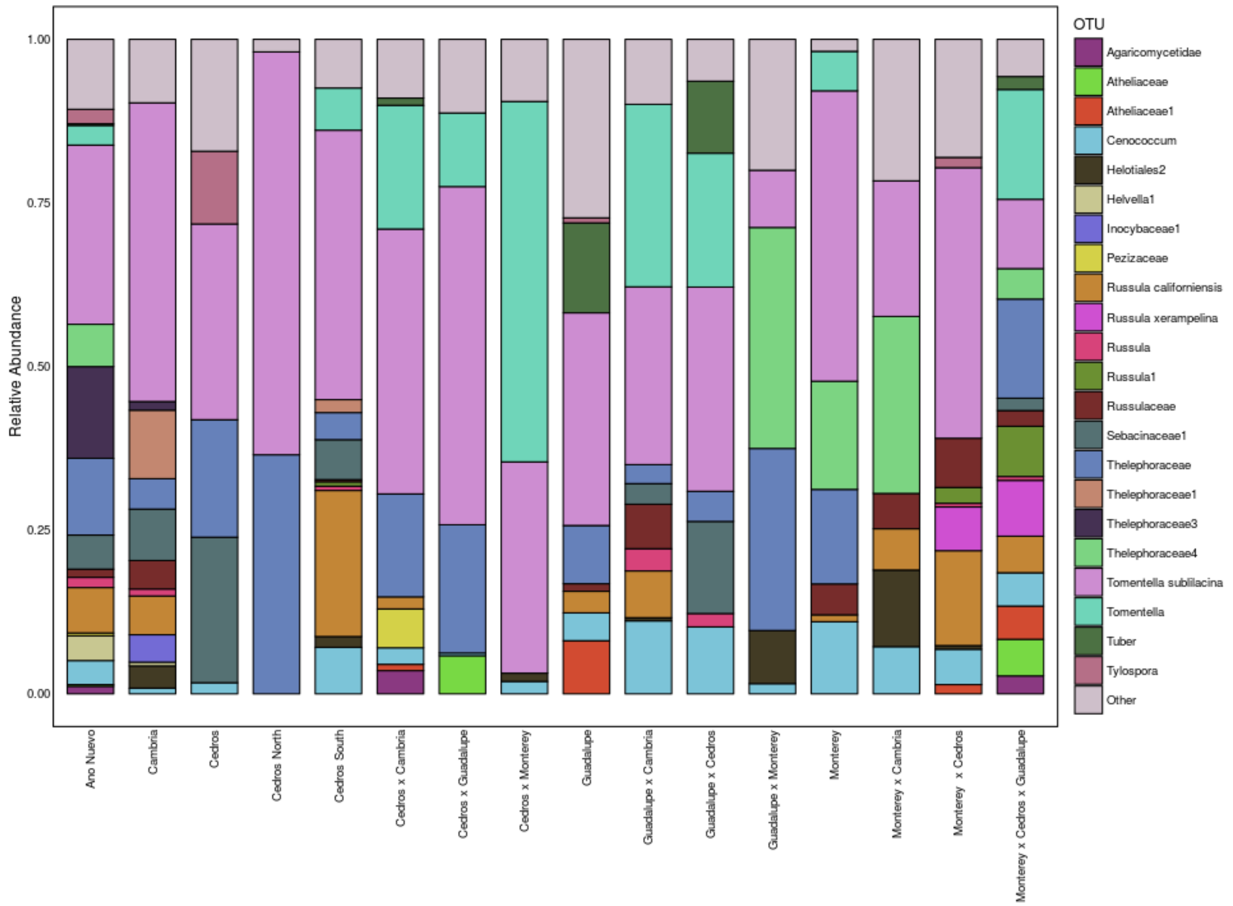

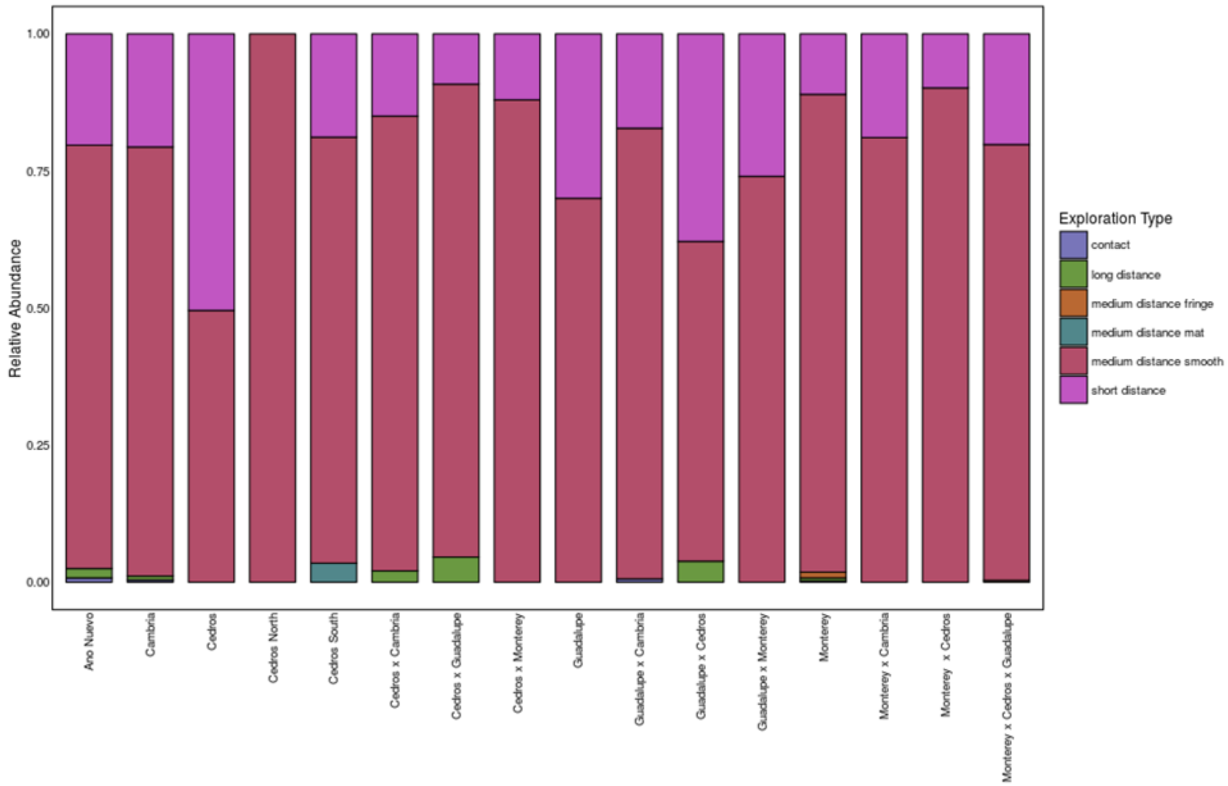

Relative Abundance
1.00
0.75
0.50
0.25
0.00
Ano Nuevo
Cambria
Cedros
Cedros North
Cedros South
Cedros x Cambria
Cedros x Guadalupe
Cedros x Monterey
Guadalupe
Guadalupe x Cambria
Guadalupe x Cedros
Guadalupe x Monterey
Monterey
Monterey x Cambria
Monterey x Cedros
Monterey x Cedros x Guadalupe
Exploration Type
contact
long distance
medium distance fringe
medium distance mat
medium distance smooth
short distance

Figure S3: Relative abundances of ectomycorrhizal fungi by fungal hyphal biomass recovered from seedlings, across Monterey pine genetic backgrounds planted in a common garden experiment. Relative abundance was calculated as the proportion of sequences belonging to a particular lineage of all ITS gene sequences recovered from each seedling. Sequences were pooled by seedling genetic background.

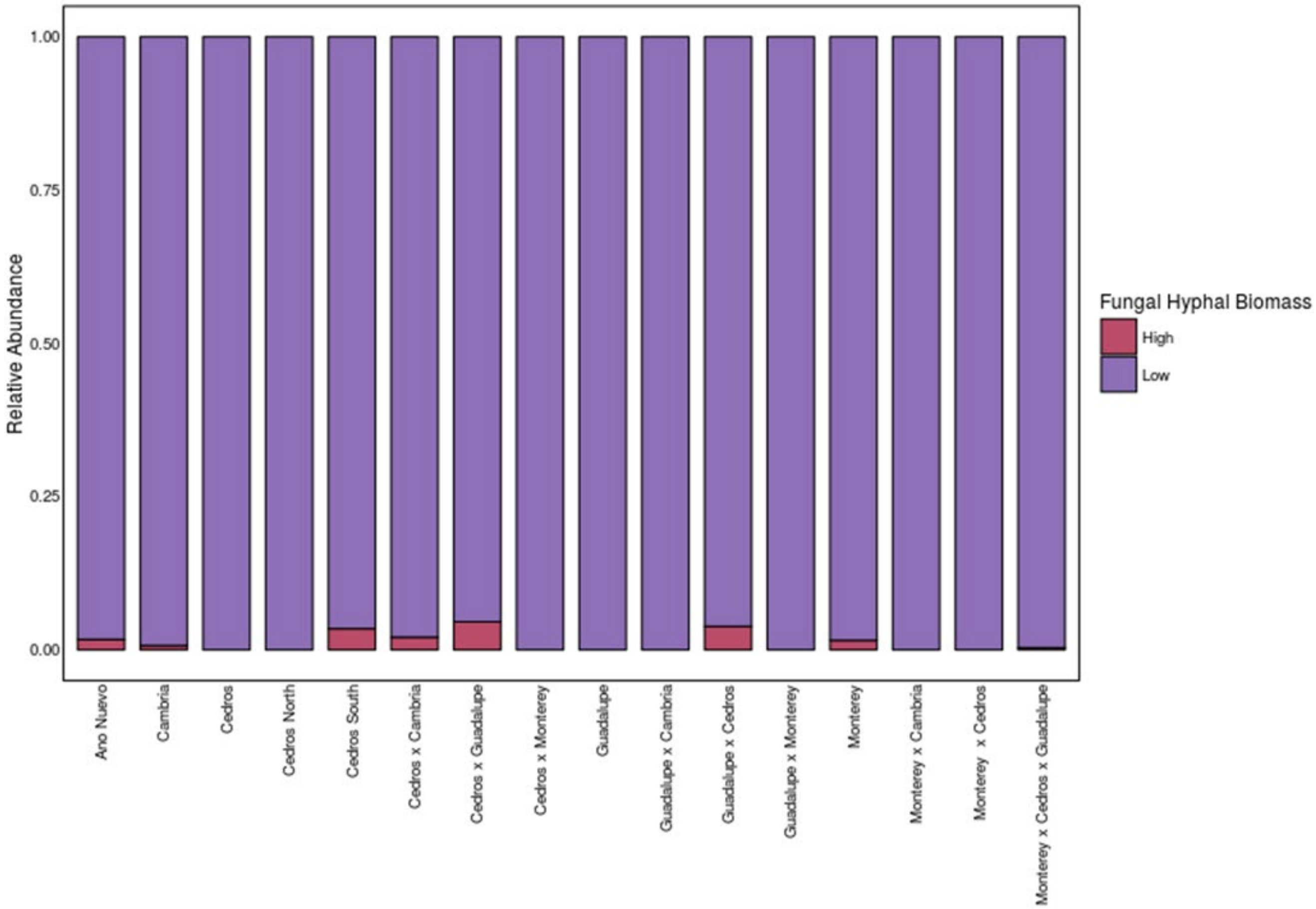

Supplementary Material C: Methods

Field experiment and measured traits

Seeds representing single populations were directly collected from those populations from 2002-2006 'Mexus'[1,2]. Seeds from controlled crosses were collected from a series of experiments previously established by W. Libby & colleagues in the 1960s at the University of California, Berkeley's Russell Research Station ('Russel Reserve)[3] in Orinda, California (37.918, -122.158). To obtain intermediate phenotypes/genotypes, we included open-pollinated F2 offspring from factorial crosses of the adult progeny from each native population. Additionally, we included offspring from Monterey/Cedros crosses that were further crossed with one mainland (Año Nuevo) and one island population (Guadalupe) to create crosses with genetic information from three populations (Monterey / Cedros x Año, Monterey / Cedros x Guadalupe).

Laboratory processing of root samples and morphotyping

Roots from each harvested seedling were hand washed over a 2 mm sieve to remove rhizosphere soil, and were examined using a dissecting microscope. The number of root tips with viable ECM fungal colonization was counted, and each ECM root tip was classified into a morphotype based on morphological distinctions such as color, texture, branching patterns, and emanating hyphae or rhizomorphs. Two root tips per morphotype observed in each sample were removed for identification via Sanger sequencing. All laboratory processing of root samples and morphotyping occurred within 21 days of collection.

DNA extraction and sequencing

After morphotyping, DNA was immediately extracted from two root tips per sample using components of a Sigma Extract-N-Amp extraction kit (Sigma-Aldrich, St. Louis, MO). 10 μL of the Sigma Extraction Buffer was added to each root tip, heated to 65°C for 10 minutes,

95°C for 10 minutes, and then 30 µL of Sigma Neutralization Solution and 60 µL PCR-grade water was added. Samples were then stored at -20°C for approximately one month.

To facilitate Sanger sequencing of ECM fungal species colonizing root tips, the Internal Transcribed Spacer (ITS) region of the fungal nuclear genome was amplified using the fungal-specific forward and reverse primers, ITS1-F and ITS4[4]. Amplification was achieved according to the protocol outlined in Rúa et al.[5] and success was checked on a 1 % agarose gel with SYBR® Safe DNA gel stain (Molecular Probes, Eugene, OR, USA). Approximately 10% of samples did not amplify with the default settings. The raw DNA for these samples was diluted to 1% DNA (1 µl DNA + 99 µl sterile PCR-grade water) and amplification reactions were repeated using the same methods as the default except that the number of cycles for denaturation, annealing, and extension was increased from 35 cycles to 40 cycles. Approximately 5% of dilutions did not amplify with these settings so amplification was repeated with the same methods as the previous dilutions and the annealing temperature was lowered from 52°C to 51°C.

Excess primer and unincorporated nucleotides were removed enzymatically using the Exonuclease I (ExoI) and Antarctic Phosphotase (AP) enzymes (New England BioLabs, Inc., Ipswich, MA, USA) with the following procedure: 0.5 µl ExoI, 0.5 µl AP, and 4.5 µl sterile PCR-grade water were added to 5 µl of the PCR product and incubated at 37°C for 45 minutes, then 80°C for 20 minutes, and finally 4°C for at least 5 minutes.

Sanger sequencing was performed using the forward primer ITS5[6] and the Big Dye Terminator Sequencing Kit (v3.1, Invitrogen Corp.). Although unidirectional sequencing of the ITS region in fungi may result in a higher proportion of ambiguous bases and the potential exclusion of some OTUs from the data, bidirectional sequencing was not feasible for our study,

due to the large number of samples examined. Each Big Dye reaction contained 0.4 μL Big Dye Reaction Premix, 1.8 μL Big Dye 5 X sequencing buffer, 0.5 μL of the forward primer at 10 μM concentration, 6.3 μL of PCR-grade water, and 1 μL of the cleaned PCR product. Amplification conditions were 96^0C for 1 minute; followed by 35 cycles of 95^0C for 30 seconds, 50^0C for 20 seconds, and 60^0C for 4 minutes. Reactions were dried and shipped overnight to the DNA Lab at Arizona State University, in Tempe, Arizona, where the Big Dye reactions were purified and read on an Applied Bioscience 3730 capillary genetic analyzer.

Editing

The fungal DNA sequences were edited manually in Geneious software (Dotmatics, Boston, MA, USA), correcting ambiguous bases associated with dye blobs and elsewhere when possible. All sequences with >3% ambiguous bases or < 200 base pairs long were deleted. Remaining sequences were subjected to OTU assembly (at 97% similarity) using CAP3 software[7] on the University of Alaska, Fairbanks (UAF) Life Science Informatics server, using default settings except the following: maximum overhang percent length = 60, match score factor = 6, overlap percent identity cut-off = 97, clipping range = 6, as described previously[5].

Taxonomic assignment of sequences

Consensus fungal sequences from each OTU were checked using BLAST[8] searches on the International Nucleotide Sequence Database (INSD) and the User-Friendly Nordic ITS Ectomycorrhizal (UNITE) database[9] to obtain best matches for taxonomic affiliation of OTUs. The ultimate decision on the best match to a sequence was based on both similarity and length of the match. Sequences >97% similar in composition to database sequences from named, cultured fungi were considered the same OTU (hereafter, ‘species’). Sequences with matches showing 94-97% similarity to a database sequence with an assigned species epithet, or matching a sequence

identified only to genus, were assigned into the respective genus and assigned a number (e.g., *Russula* 1). Similarly, those matches in the database <94%, but greater than 90% were assigned to the appropriate taxonomic family. Any matches <90% similar to database sequences were excluded. If sequence matches among the two sequence repositories showed equal affinity or similarity to multiple genera within a family, priority was given to the vouchered specimens residing on the UNITE database. Any species known to be strictly non-mycorrhizal was eliminated from the data set. The raw fungal sequence data for this project have been submitted to the GenBank databases under the accession numbers MN364462 - MN364644.

Statistical analyses

Quantitative Genetics

i. Heritability

To test model terms and estimate means and co(variance) components for traits, we used univariate linear models (plant traits, diversity indices) and generalized models with the Poisson (ECM fungal tip abundance) and negative binomial (ECM fungal exploration types, fungal biomass) fit with the *lmer* or *glmer* functions from the *lme4* package [10] following the animal model[11]. Each model fit the desired trait as a function of a model constant (μ), random effects for family (*n* = 39) and population (*n* = 16), and the residual random effect (*n* = 428).

We then computed narrow-sense heritability (h^2) on the observed scales from these models using the *QGparams* function of the *QGglmm* package[12]. The latent heritability was then estimated as $h^2 = V_A/V_P$ where V_A is the additive genetic variance and V_P is the phenotypic variance (i.e., the sum of the variance components). The reliability of these estimates was assessed using 95% credible intervals calculated on posterior distributions of the models fit using

the function *MCMCglmm* from the *MCMCglmm* package using weakly informed priors appropriate to the model types described above[12,13].

ii. Genetic variance-covariance (G) matrix

Genetic variances of mycorrhizal traits were estimated from the plant genetic families component of an analysis of variance in which traits were the dependent variables and plant genetic family was the independent variable. Significance of the plant family effect in this ANOVA was used to determine the significance of the genetic variation. Covariances of the plant family means were used as an estimate of the genetic covariances. Genetic variances and covariances were used to construct the G-matrix, a square matrix with covariances in the off-diagonal and with genetic variances in the diagonal *sensu*[14]. Different G-matrices were constructed for each set of traits as described above, with each matrix including the same four plant traits (root:shoot, diameter, RGR, biomass) and the mycorrhizal and fungal traits of interest for that particular model (OTUs, five most abundant OTUs, families, fungal exploration types and hyphal biomass, or diversity metrics).

Bootstrapping was used to determine whether covariances were significantly different from zero. First, we calculated the mean of each trait for each of the 39 plant genetic families, resulting in a set of 39 vectors with the number of elements in each vector reflective of the number of phenotypic traits in that model. For example, to determine significance of covariances for models examining plant traits and the five most abundant fungal OTUs, the number of elements in each vector would be nine, representing the plant genetic family means for the four plant and five mycorrhizal or fungal traits. A bootstrap sample was then created by randomly sampling (with replacement) 39 of these vectors. From the bootstrap sample, the genetic covariances were calculated. The bootstrapping procedure was repeated 5000 times. A two-tailed

test with an overall significance level of α = 0.05 for each covariance was used to determine the significance of each off-diagonal element of the G-matrix. Specifically, for each element, we determined the proportion of the 5000 bootstrap values that were greater than 0. The covariance between traits was considered significantly positive if more than 4875 covariance estimates were greater than 0 and significantly negative if fewer than 125 covariance estimates were greater than 0[14].

Analyses of Natural Selection

We quantified natural selection using genotypic selection analysis, which uses the family means as a unit of observation to avoid bias due to the local environmentally induced covariance between phenotypes and fitness [15,16]. All phenotypic traits were standardized to a mean of zero and a variance of one. The directional selection differential (S_i), which estimates the total strength of selection due to both direct and indirect selection, was estimated as the covariance between relative fitness and each standardized trait in separate models. Directional (linear) selection gradients (β_i) were estimated as the covariance between relative fitness and each standardized trait in a full model. Significant selection gradients indicate that selection favors either a phenotypic mean increase (if positive) or decrease (if negative). We report both differentials and gradients, but for simplicity we focus our discussion on gradients. To better understand selection for plant and mycorrhizal traits, relative frequency of the direction of selection for individual plant traits was synthesized across models for both differentials and gradients.

i. Plant Fitness

Selection on plants was estimated using three different proxies for plant fitness: relative seedling biomass, relative RGR, and seedling survival. Selection on relative seedling biomass

(individual total biomass divided by mean biomass) and relative RGR (individual RGR divided by mean RGR) was assessed via linear models with a response variable representing plant fitness, and standardized trait values (see Supplementary Material A) as explanatory variables using the *lm* function from the *stats* package[17]. Selection on seedling survival was calculated as regressions of relative survival (mean survival for each plant genetic family) onto standardized traits using logit links to account for binomial survival distributions[18]. In these analyses, trait values were assigned at the level of plant genetic families, using averages of measurements across surviving plants from each family. While general linear models were used to extract selection gradients[15], statistical significance ($P<0.05$) of each selection gradient related to seedling survival was assessed using logistic regression models due to violations of the assumptions of parametric statistics inherent in survival data[18,19].

ii. Phenotypic Traits

In the phenotypic selection models, six variables (root:shoot, diameter, SRL, RGR, biomass, and survival) were always considered plant traits, and a subset of those (RGR, biomass, and survival) were treated as proxies for plant fitness. All models included relative fitness as the dependent variable and some or all of the five standardized continuous plants traits [root:shoot ratio (all models), RGR (biomass and survival models only), biomass (RGR models only), diameter (all models), and SRL (all models)] as independent variables. Total ECM fungal tip abundance (Model 1), relative abundances of individual ECM fungal OTUs or families ('mycorrhizal traits' as in[20]; Model 2), and ECM fungal diversity metrics (Model 3) were included as traits that may be influenced by both the plant and fungal genomes (mycorrhizal traits). ECM fungal exploration types and ECM fungal biomass (‘High’ or ‘Low’) are

morphological traits of particular fungal taxa, and thus were interpreted only as traits of the whole ECM fungal community.

Separate fitness models were fit using standardized values for fungal exploration types and fungal biomass as independent variables because the fungal traits were highly correlated (all Pearson's correlations $p<0.0001$, Table S2B). Because fungal biomass had only two values ('High' or 'Low'), positive selection on this variable indicated selection for fungi with low hyphal biomass over fungi with high hyphal biomass. Supplementary Material A summarizes the suite of different selection models analyzed. Depending on the model, standardized values of total ECM tip abundance (Supplementary Material A: Models 1), values calculated for the most abundant ECM fungal OTUs and families (Supplementary Material A: Model 2), values for tip abundance calculated by fungal species identity (Supplementary Material A: Models 3), values calculated by fungal traits (Supplementary Material A: Models 4, 5) or values calculated by fungal richness (Supplementary Material A: Models 6) were used. Additional details about model structure can be found in Supplementary Material A.

iii. Interspecific selection

In selection models, interaction terms that combine plant traits and fungal traits allow for the assessment of interspecific selection, i.e., selection by one or more species on a focal species. Here, such a term represents selection on the plant trait resulting from its covariance with the fungal trait, and thus our analyses estimate selection in one of the two directions of reciprocal selection necessary to demonstrate coevolution. In those same models, main effect terms indicate direct environmental selection on traits, not mediated by plant-fungal interactions.

Interactions between plant traits and traits of the ECM fungal community were assessed in a subset of the selection models. For mycorrhizal traits potentially influenced by both the plant

and fungal genomes (total ECM tip abundance, relative abundance of particular ECM fungal OTUs or families, and diversity metrics of ECM fungi), we considered interactions with plant traits to indicate *potential* interspecific selection. For interactions between unambiguous plant traits with unambiguous fungal traits (ECM fungal hyphal biomass and exploration type), interpretation was more straightforward, providing stronger evidence for interspecific selection on traits of Monterey pine by traits of its ECM fungal community. In the "basic" model structure, we assessed interactions between the focal plant traits and total ECM tip abundance (Supplementary Material A: Model 1). In the "most abundant OTUs" model structure, we assessed interactions between the focal plant traits and the relative abundances of fungal OTUs *Cenococcum*, *Russula californiensis*, Sebacinaceae1, Thelephoraceae1, *Tomentella1*, and *Tomentella sublilacina* (Supplementary Material A: Model 2a-c). In the "most abundant families" model structure, we assessed interactions between the focal plant traits and the relative abundances of the fungal families Gloniaceae, Russulaceae, Sebacinaceae, and Thelephoraceae (Supplementary Material A: Model 2d-f). In the "fungal hyphal biomass" model structure, we assessed interactions between the focal plant traits and the ECM fungal community trait fungal hyphal biomass (Supplementary Material A: Model 4). In the "exploration type" model structure, we assessed interactions between the focal plant traits and the ECM fungal community trait fungal exploration type (Supplementary Material A: Model 5). In "alpha diversity" models, we assessed interactions between the focal plant traits and ECM fungal Shannon and Observed alpha diversity (Supplementary Material A: Model 6). In order to broaden the number of fungal OTUs and families we could assess for phenotypic selection, we also fit a set of models with only the focal plant traits and fungal OTUs/families without interactions (Supplementary Material A: Model 3).

iv. Fitness landscapes

The shapes of fitness surfaces reflect the contributions of the various underlying fitness components to overall fitness; in this case, the contribution of plant and fungal traits to plant fitness. To visualize interspecific selection, we illustrated selection surfaces for traits with significant plant x fungal interactions in total selection models (*S*) using thin plate splines, as they allow for minimal assumptions regarding the structure of the data. Relevant trait values were extracted from total selection models using the *predict* function from the *stats* package[17] and fit to a thin plate spline surface with relative fitness values calculated as described above using the *Tps* function from the *fields* package[21] The resulting fitness landscapes were then illustrated with the *drape.plot* function from the *fields* package. For consistent comparison, rotation angles of each panel are the same.

Seedling Survival

To test for evidence of local adaptation (home-field advantage) in the Cambria population of Monterey pine, adaptation by mainland versus island populations, and advantage of hybrid versus single population genetic background, the odds of overall seedling mortality were modeled as a function of host genetic background as described by Cambria genotype (Cambria vs non-Cambria), location (Mainland vs Island vs Mixed-Background), and hybridization (Pure Background vs Hybrid), using logistic regression via the *glm* function from the *stats* package[17].

References

1. Hoeksema, J. D. & Thompson, J. N. Geographic structure in a widespread plant–mycorrhizal interaction: pines and false truffles. *Journal of Evolutionary Biology* **20**, 1148–1163 (2007).

2. Hoeksema, J. D., Hernandez, J. V., Rogers, D. L., Mendoza, L. L. & Thompson, J. N. Geographic divergence in a species-rich symbiosis: Interactions between Monterey pines and ectomycorrhizal fungi. *Ecology* **93**, 2274–2285 (2012).
3. Burdon, R. & Libby, W. J. *Genetically Modified Forests: From Stone Age to Modern Biotechnology*. (Duke University Press, 2007).
4. Gardes, M. & Bruns, T. D. ITS primers with enhanced specificity for basidiomycetes---application to the identification of mycorrhizae and rusts. *Molecular Ecology* **2**, 113–118 (1993).
5. Rúa, M. A. *et al.* Associations between ectomycorrhizal fungi and bacterial needle endophytes in Pinus radiata: implications for biotic selection of microbial communities. *Frontiers in Microbiology* **7**, (2016).
6. White, T. J., Bruns, T., Lee, S. & Taylor, J. W. Amplification and direct sequencing of fungal ribosomal RNA genes for phylogenetics. in *PCR Protocols: A Guide to Methods and Applications* (eds. Innis, M. A., Gelfand, D. H., Sninsky, J. J. & White, T. J.) 315–322 (Academic Press, Inc., New York, 1990).
7. Huang, X. & Madan, A. Cap3: A DNA sequence assembly program. *Genome Research* **9**, 868–877 (1999).
8. Altschul, S. F., Gish, W., Miller, W., Myers, E. W. & Lipman, D. J. Basic local alignment search tool. *Journal of Molecular Biology* **215**, 403–410 (1990).
9. Kõljalg, U. *et al.* Towards a unified paradigm for sequence-based identification of fungi. *Molecular Ecology* **22**, 5271–5277 (2013).
10. Bates, D., Maechler, M., Bolker, B. & Steve, Walker. Fitting Linear Mixed-Effects Models Using lme4. *Journal of Statistical Software,* **67**, 1–48 (2015).

11. Wilson, A. J. *et al.* An ecologist's guide to the animal model. *Journal of Animal Ecology* **79**, 13–26 (2010).

12. de Villemereuil, P., Schielzeth, H., Nakagawa, S. & Morrissey, M. General Methods for Evolutionary Quantitative Genetic Inference from Generalized Mixed Models. *Genetics* **204**, 1281–1294 (2016).

13. Hadfield, J. D. MCMC Methods for Multi-Response Generalized Linear Mixed Models: The MCMCglmm R Package. *Journal of Statistical Software* **33**, 1–22 (2010).

14. Wise, M. J. & Rausher, M. D. Evolution of resistance to a multiple-herbivore community: genetic correlations, diffuse coevolution, and constraints on the plant's response to selection. *Evolution* **67**, 1767–1779 (2013).

15. Lande, R. & Arnold, S. J. The Measurement of Selection on Correlated Characters. *Evolution* **37**, 1210–1226 (1983).

16. Rausher, M. D. The measurement of selection on quantitative traits: biases due to environmental covariances between traits and fitness. *Evolution* **46**, 616–626 (1992).

17. R Core Team. *R: A Language and Environment for Statistical Computing.* https://www.R-project.org/ (2023).

18. Janzen, F. J. & Stern, H. S. Logistic Regression for Empirical Studies of Multivariate Selection. *Evolution* **52**, 1564–1571 (1998).

19. Calsbeek, R. & Irschick, D. J. The quick and the dead: correlational selection on morphology, performance, and habitat use in island lizards. *Evolution* **61**, 2493–2503 (2007).

20. Piculell, B. J., José Martínez-García, P., Nelson, C. D. & Hoeksema, J. D. Association mapping of ectomycorrhizal traits in loblolly pine (Pinus taeda L.). *Molecular Ecology* **28**, 2088–2099 (2019).

21. Nychka, D., Furrer, R., Paige, J. & Sain, S. fields: Tools for spatial data. *R package version 9.6* (2017) doi:10.5065/D6W957CT.

Supplementary Material D: Results

G matrix

Of the 351 covariances calculated between plant traits and fungal OTUs, 141 were nominally significant at the $P < 0.05$ level (Table S8). At an overall significance level of 0.05, at most 30 of these would be expected to be found significant by chance alone. Consequently, at least 111 of the 141 covariances are "truly significant" (not counting covariances found to be nonsignificant due to Type II error). Of the 141 nominally significant covariances, 136 were negative and 5 were positive. Of the 36 covariances calculated between plant traits and fungal exploration types, 26 were nominally significant at the $P < 0.05$ level (Table S8). At an overall significance level of 0.05, at most three of these would be expected to be significant by chance alone. Consequently, at least 23 of the 36 covariances are truly significant. All of the 26 nominally significant covariances were negative.
