# Supplementary figures and images for: Interspecific selection in a diverse mycorrhizal symbiosis
Source: Sci Rep. 2024 May 27;14:12151. doi: 10.1038/s41598-024-62815-4 (PMC11130337; doi:10.1038/s41598-024-62815-4)

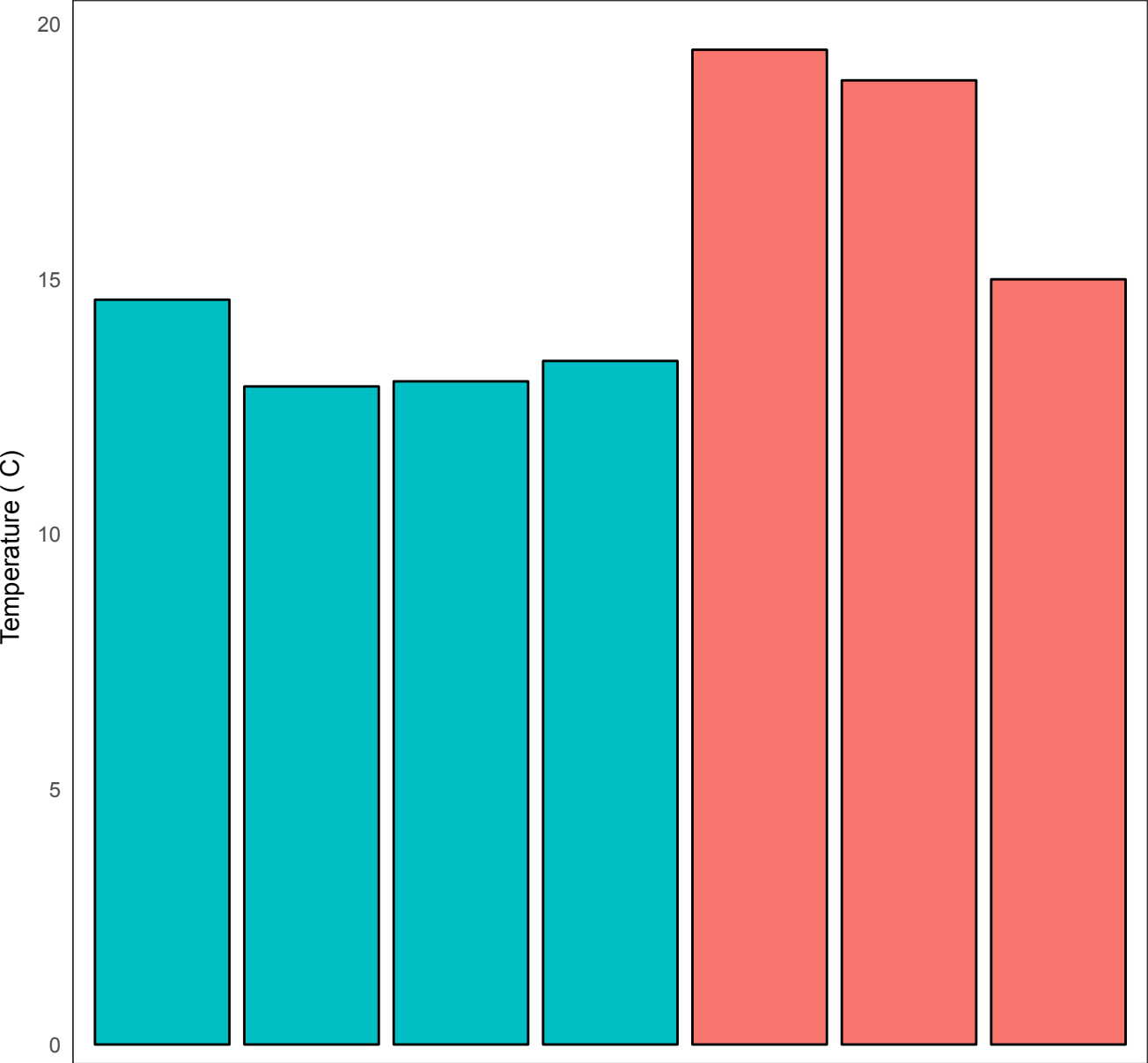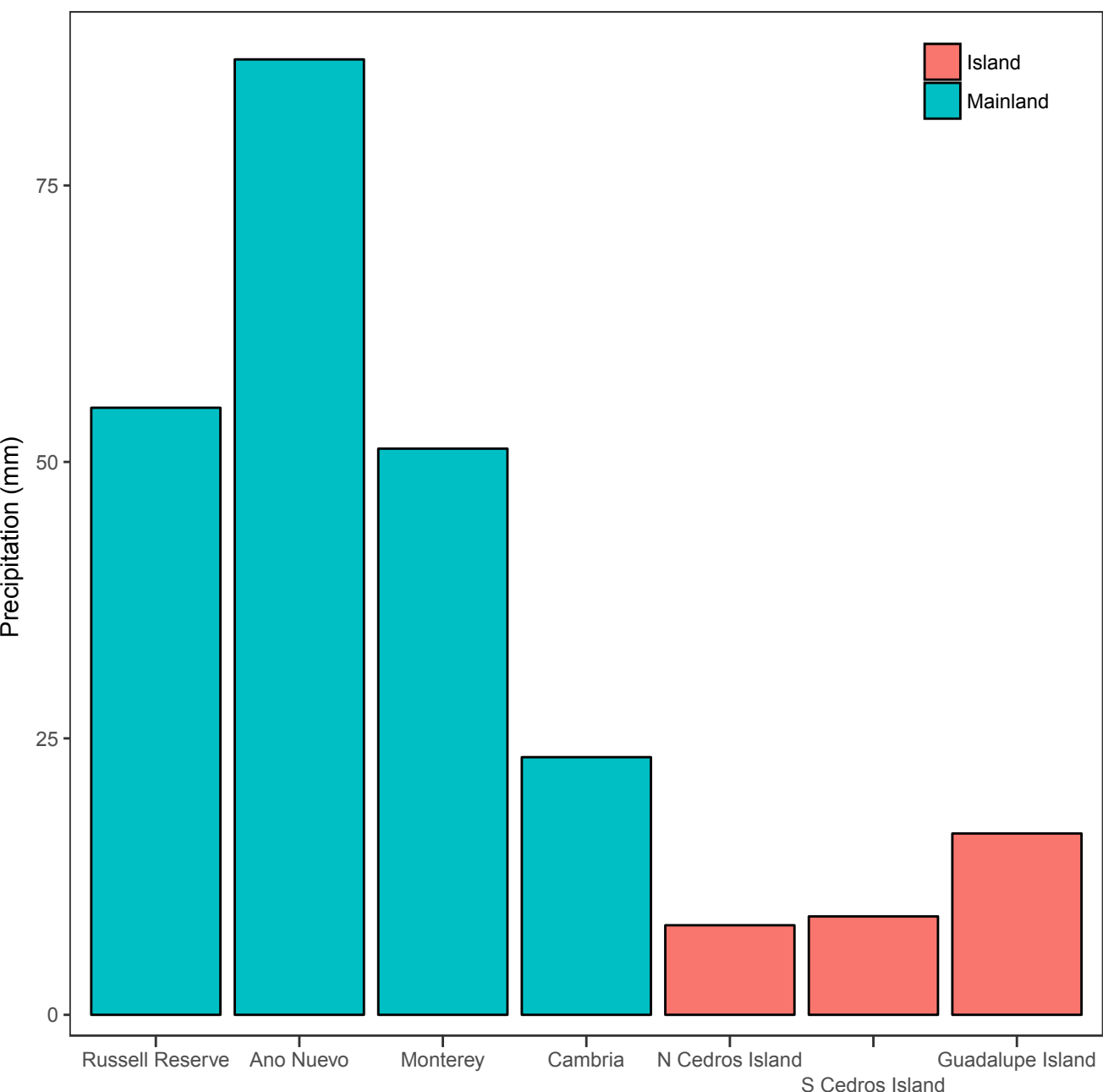

Supplement: Supplementary file 2 — Supplementary Information 2. [file 41598_2024_62815_MOESM2_ESM.pdf]

a)

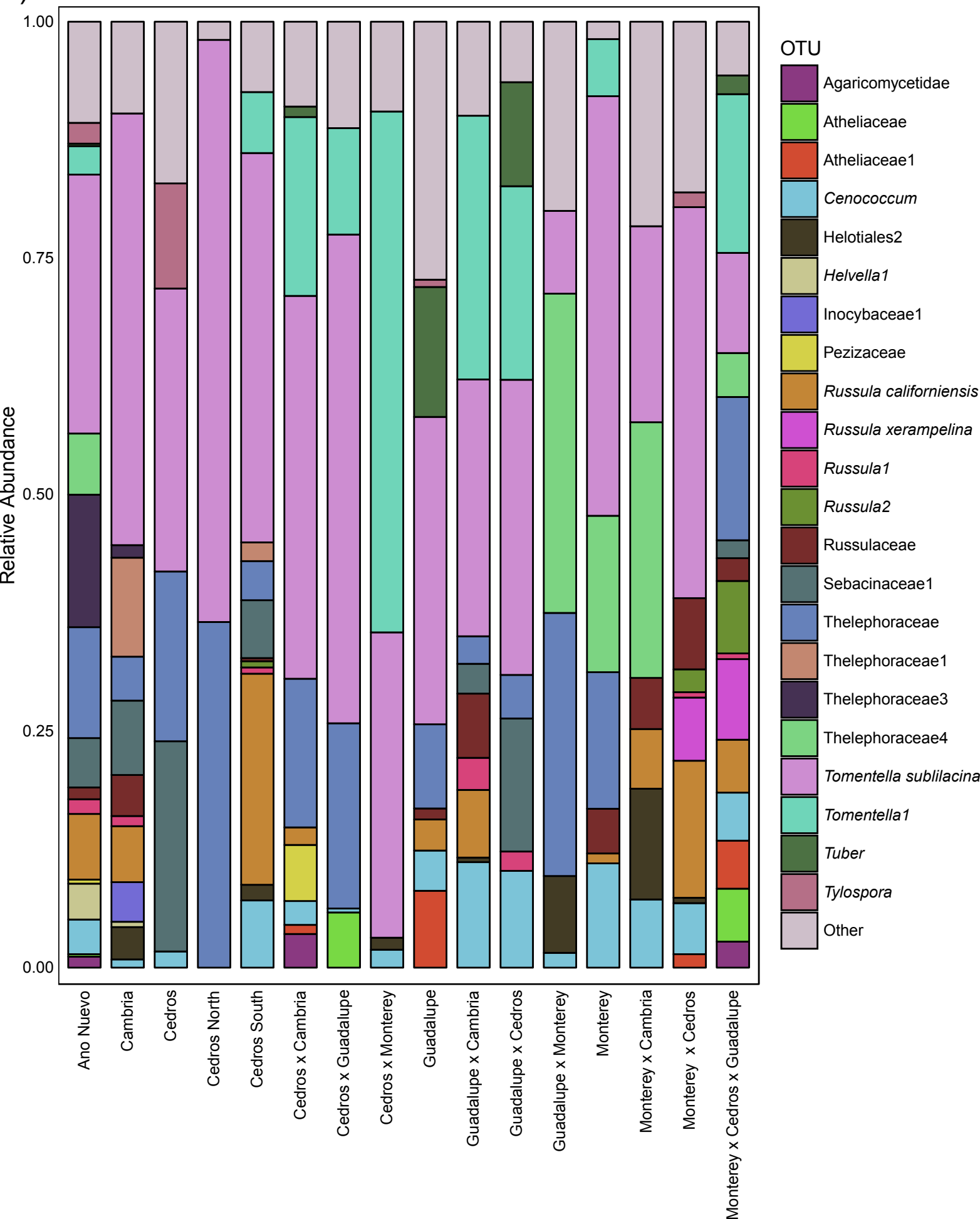

Supplement: Supplementary file 3 — Supplementary Information 3. [file 41598_2024_62815_MOESM3_ESM.pdf]

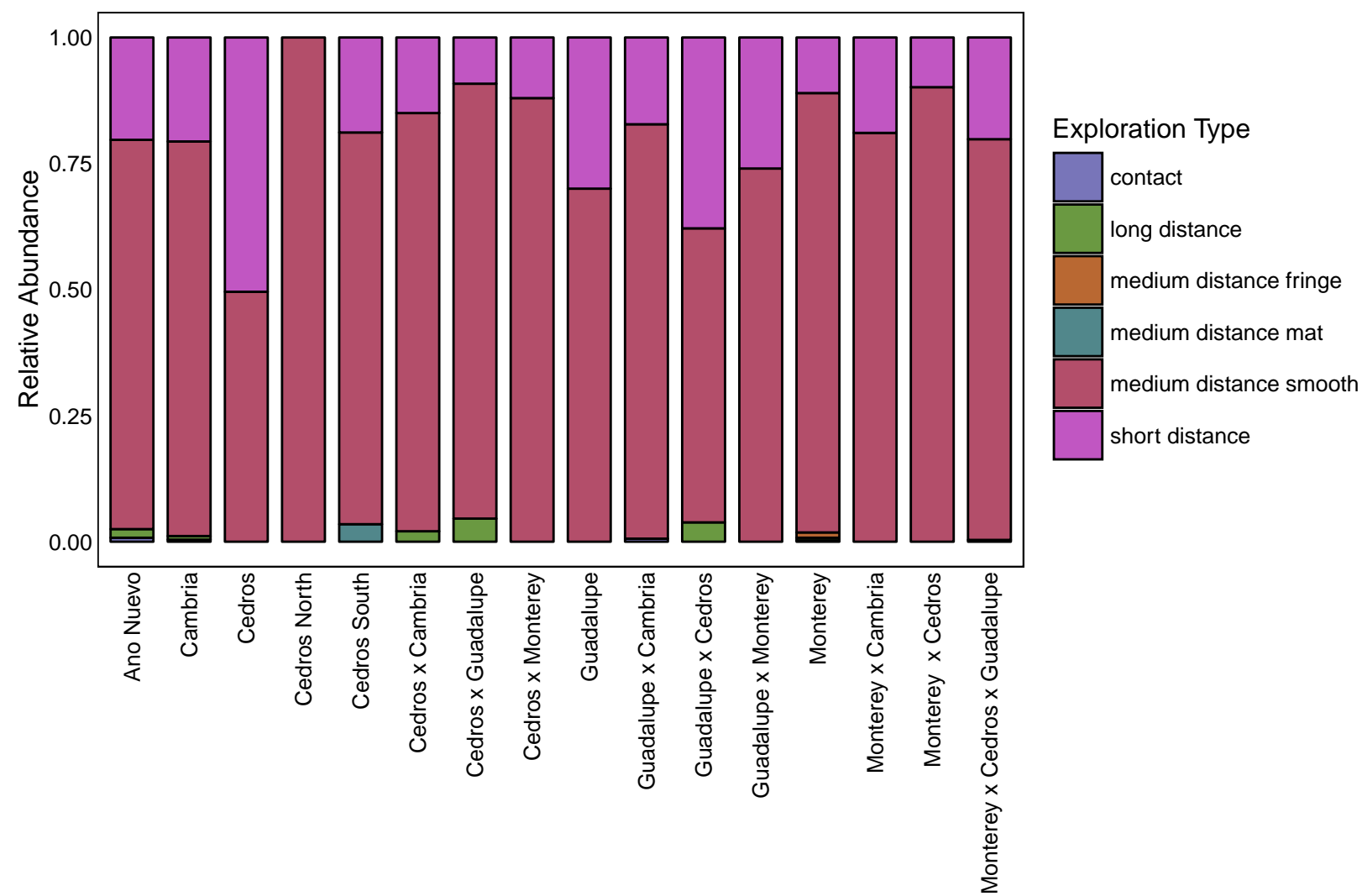

Supplement: Supplementary file 4 — Supplementary Information 4. [file 41598_2024_62815_MOESM4_ESM.pdf]

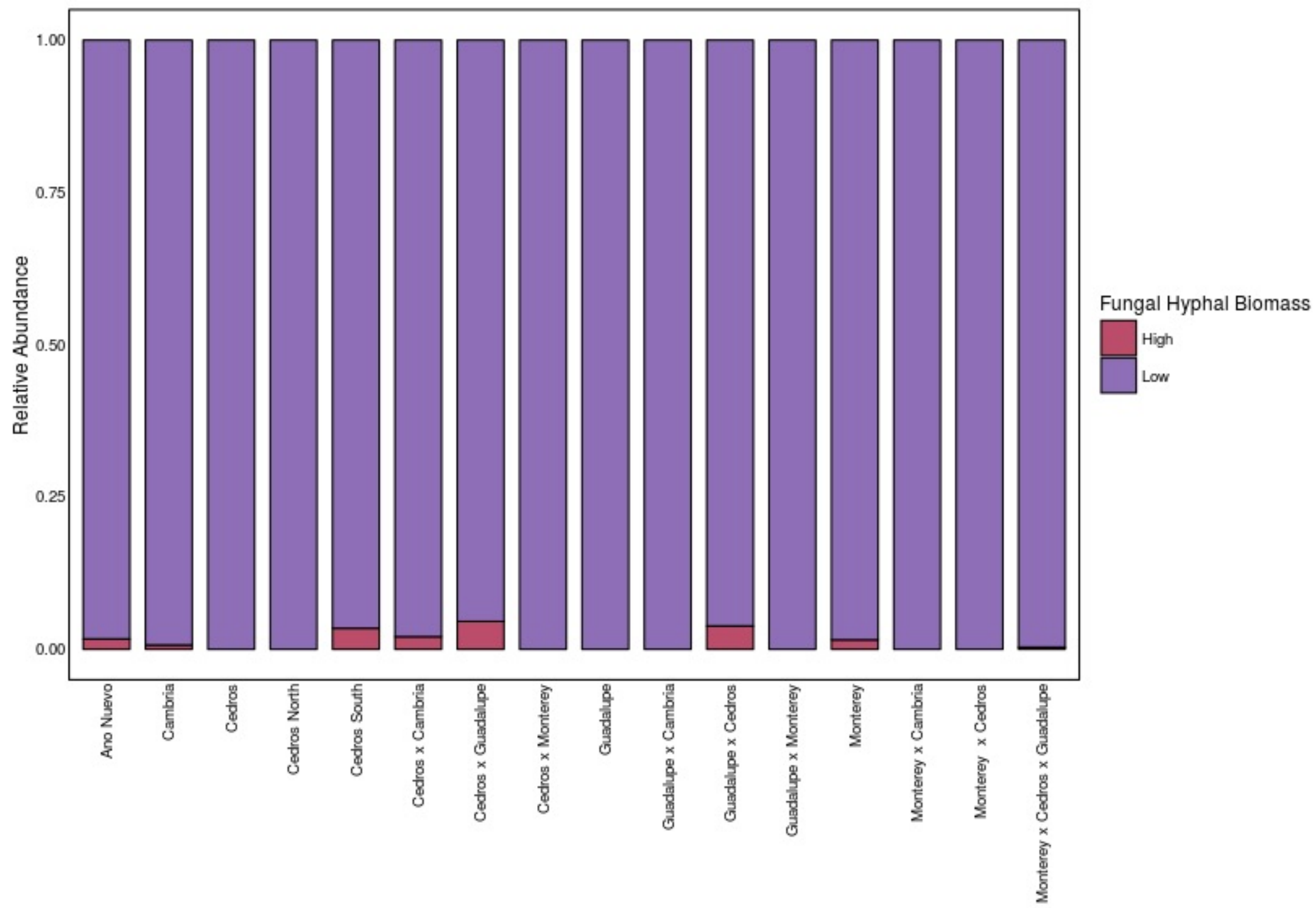

Supplement: Supplementary file 5 — Supplementary Information 5. [file 41598_2024_62815_MOESM5_ESM.pdf]
