# Supplementary material for: Interspecific selection in a diverse mycorrhizal symbiosis
Source: Sci Rep. 2024 May 27;14:12151. doi: 10.1038/s41598-024-62815-4 (PMC11130337; doi:10.1038/s41598-024-62815-4)
Supplement: Supplementary file 6 — Supplementary Information 6. [file 41598_2024_62815_MOESM6_ESM.pdf]

Table S8. Genetic variance–covariance matrix (G-matrix) for operational taxonomic units (OTUs) and plant t
Genetic variances are on the main diagonal and covariances are off-diagonal elements.
Genetic variances and covariances significant at $P < 0.05$ indicated in bold.
Heritability (h^2) and 95% credible interval (CI) is reported in the first column.

	h^2 (95% CI)	Biomass	Diam	RGR	RS	Agaricomycetidae	Atheliaceae1	Atheliaceae2
Biomass	0.22 (0.11,0.37)	**-0.095**	**0.114**	**0.317**	-0.061	0.030	-0.010	0.026
Diam	0.22 (0.09,0.36)		**-0.047**	0.100	0.037	0.017	0.039	0.007
RGR	0.264 (0.14,0.40)			**-0.136**	-0.080	0.018	-0.053	0.028
RS	0.06 (0.008,0.15)				**-0.053**	-0.002	0.004	0.030
Agaricomycetidae	0.135 (0.07,0.21)					-0.027	**-0.003**	**-0.003**
Atheliaceae1	0.23 (0.11,0.3)						-0.009	0.026
Atheliaceae2	0.175 (0.08,0.25)							-0.031
Cenococcum	0.05 (0.02,0.11)							
Helotiales2	0.148 (0.07,0.24)							
Helvella1	0.07 (0.03,0.13)							
Inocybaceae1	0.09 (0.04,0.16)							
Pezizaceae	0.053 (0.03,0.13)							
Russula1	0.14 (0.07,0.22)							
Russula californiensis	0.12 (0.05,0.20)							
Russula xerampelina	0.183 (0.10,0.28)							
Russula2	0.06 (0.02,0.11)							
Russulaceae	0.128 (0.06,0.21)							
Sebacinaceae1	0.156 (0.08,0.25)							
Thelephoraceae	0.105 (0.04,0.19)							
Thelephoraceae1	0.104 (0.03,0.19)							
Thelephoraceae3	0.066 (0.02,0.12)							
Thelephoraceae4	0.149 (0.07,0.23)							
Tomentella4	0.343 (0.2,0.5)							

Tomentella1	0.139 (0.06,0.23)							
Tomentella sublilacina	0.05 (0.02,0.09)							
Tuber	0.168 (0.09,0.26)							
Tylospora1	0.126 (0.06,0.21)							

R : S = Root (mg) : Shoot (mg); RGR = relative growth rate

traits.

Cenococcum	Helotiales2	Helvella1	Inocybaceae 1	Pezizaceae	*Russula1*	*Russula californiensis*	*Russula xerampelina*	*Russula2*	Russulaceae
0.054	0.081	0.006	**-0.012**	0.072	-0.030	0.028	0.004	0.025	**0.124**
0.009	-0.036	0.053	0.010	0.003	-0.025	0.010	0.008	0.003	0.082
0.033	0.037	0.031	**-0.010**	0.048	-0.078	**0.078**	0.027	0.023	**0.130**
0.022	-0.051	-0.008	0.050	-0.012	-0.031	0.000	**-0.017**	0.038	-0.032
**-0.013**	**-0.006**	**-0.003**	**-0.002**	**-0.003**	**-0.007**	0.005	**-0.002**	**-0.002**	**-0.008**
-0.010	**-0.012**	**-0.006**	**-0.004**	**-0.006**	**-0.015**	**-0.020**	**-0.004**	0.025	**-0.018**
0.020	**-0.011**	**-0.006**	**-0.003**	**-0.006**	**-0.014**	0.009	**-0.004**	0.048	**-0.017**
-0.028	0.006	0.010	0.001	0.022	0.101	0.006	0.004	0.030	-0.019
	**0.015**	**-0.012**	**-0.007**	**-0.012**	0.028	-0.004	**-0.008**	-0.006	0.000
		-0.013	**-0.004**	**-0.006**	**-0.015**	0.028	**-0.004**	**-0.005**	0.001
			-0.019	**-0.004**	0.015	-0.005	**-0.002**	**-0.003**	**-0.010**
				-0.013	-0.009	**-0.019**	**-0.004**	**-0.005**	**-0.018**
					0.019	**-0.036**	**-0.010**	**-0.011**	**-0.043**
						-0.062	0.025	-0.010	0.035
							-0.013	**-0.003**	0.016
								-0.036	-0.005
									0.031

Sebacinaceae1	Thelephoraceae	Thelephoraceae1	Thelephoraceae3	Thelephoraceae4	Tomentella4	Tomentella1	Tomentella sublilacina	Tuber	Tylospora1
-0.013	-0.129	-0.017	**-0.031**	0.041	0.012	0.026	-0.049	0.021	-0.026
0.089	-0.169	0.008	-0.024	-0.043	0.070	-0.001	-0.010	0.011	0.038
0.006	-0.157	0.009	**-0.035**	0.064	0.051	0.036	-0.057	0.005	-0.028
0.053	0.084	-0.002	0.040	-0.067	-0.052	0.047	-0.025	0.000	0.026
**-0.008**	-0.007	**-0.003**	**-0.003**	**-0.005**	**-0.004**	0.031	**-0.011**	0.023	0.014
-0.008	-0.027	**-0.006**	**-0.006**	**-0.012**	**-0.009**	0.001	-0.007	0.016	0.026
-0.001	0.010	**-0.005**	**-0.005**	**-0.011**	0.017	0.014	**-0.028**	0.028	**-0.015**
-0.014	**-0.104**	**-0.024**	-0.013	-0.045	-0.027	0.028	**-0.100**	0.010	**-0.063**
-0.029	0.017	**-0.011**	**-0.011**	**-0.022**	**-0.017**	-0.025	-0.054	**-0.015**	**-0.032**
0.045	**-0.038**	**-0.005**	**-0.006**	**-0.011**	**-0.009**	**-0.015**	-0.004	**-0.008**	**-0.015**
**-0.009**	-0.005	**-0.003**	0.054	**-0.007**	**-0.005**	**-0.009**	0.014	**-0.005**	**-0.009**
-0.012	-0.029	**-0.005**	**-0.006**	**-0.011**	-0.002	**-0.015**	0.015	**-0.008**	**-0.016**
**-0.036**	-0.047	**-0.014**	0.019	-0.022	-0.017	-0.004	-0.008	-0.001	**-0.039**
-0.001	-0.059	0.033	0.021	0.061	-0.014	0.010	-0.033	0.005	**-0.041**
**-0.011**	**-0.025**	**-0.004**	**-0.004**	**-0.007**	**-0.006**	**-0.010**	0.007	**-0.005**	**-0.010**
0.006	-0.009	**-0.005**	**-0.005**	-0.007	**-0.007**	0.027	-0.022	0.008	**-0.013**
-0.027	**-0.104**	**-0.016**	**-0.017**	0.098	**-0.025**	-0.031	0.034	-0.010	-0.027
0.002	**-0.064**	0.018	**-0.015**	**-0.030**	-0.020	-0.011	-0.055	**-0.021**	0.212
	**0.108**	0.005	-0.004	-0.028	-0.034	-0.033	-0.123	-0.013	-0.052
		-0.021	**-0.005**	**-0.010**	**-0.008**	**-0.013**	-0.003	**-0.007**	**-0.014**
			-0.017	0.011	**-0.008**	0.016	-0.006	**-0.007**	**-0.015**
				**0.036**	**-0.016**	-0.016	-0.006	-0.011	0.037
					0.004	**-0.021**	-0.014	0.006	**-0.023**

						0.004	-0.031	0.026	-0.024
							0.001	-0.020	-0.036
								-0.038	-0.002
									0.032
